# Artificial intelligence research in school leadership and management: a text mining-based computational literature review and future directions

**DOI:** 10.3389/frma.2026.1917781

**Published:** 2026-08-12

**Authors:** Turgut Karaköse, Orhun Kaptan, Gülenay Nagihan Kılıç, İbrahim Kocabaş, Ramazan Yirci

**Affiliations:** 1 Faculty of Education, Kütahya Dumlupinar University, Kütahya, Türkiye; 2 Bakirköy Science and Art Center, Ministry of National Education (MoNE), Istanbul, Türkiye; 3 Faculty of Education, Fatih Sultan Mehmet Vakif University, Istanbul, Türkiye; 4 Faculty of Education, Yildiz Technical University, Istanbul, Türkiye; 5 Faculty of Education, Sutcuimam University, Kahramanmaraş, Türkiye

**Keywords:** artificial intelligence, leadership, school leadership, semantic networks, structural topic modeling, text mining

## Abstract

As artificial intelligence (AI) becomes increasingly embedded in school leadership and management literature, it is important to understand not only which themes are emerging, but also how coherent, mature, and distinct these themes have become. To address this need, this study conducted a text-mining-based computational literature review of 112 articles published between 2021 and 2026. The corpus was analyzed through three complementary procedures: Structural Topic Modeling (STM), conceptual maturity analysis, and semantic network fragmentation analysis. The STM analysis yielded five thematic axes: AI readiness, adoption, and self-efficacy; ethical and data-informed AI decision-making; AI literacy and digital leadership; teacher leadership and distributed agency in AI-integrated teaching; and governance frameworks for ethical and human-centered AI leadership. Ethical and data-informed decision-making exhibited the highest expected topic proportion and conceptual maturity, while AI readiness and adoption displayed moderate maturity and temporal continuity. By contrast, AI literacy and digital leadership, teacher leadership and distributed agency, and human-centered governance remained less consolidated. The semantic network analysis indicated that, within the controlled concept dictionary, the selected core concepts formed a connected co-word structure rather than a fully fragmented one; however, this pattern is interpreted as semantic convergence among dictionary-defined concepts rather than as evidence of complete theoretical integration. Future research should therefore strengthen conceptual and empirical work on these emergent themes, while extending mature themes through longitudinal, comparative, and intervention-oriented designs.

## 1 Introduction

Artificial intelligence (AI) and generative AI are fundamentally reshaping the learning and teaching processes in schools (Adams and Thompson, 2025). The field of school leadership and management has also been inevitably affected by this transformation; indeed, Fullan et al. (2024) liken the change brought about by Industry 4.0 to a tsunami. Digital transformation in education has enabled the collection of vast amounts of data from various stakeholders, and educational administrators have likewise been expected to draw on this data in their decision-making. However, the datasets collected are so large, multilevel, processual, contextual, and interactive that they cannot be evaluated with the naked eye (Arar et al., 2025). The introduction of AI applications has become an integral part of school leaders' decision-making, as these applications accelerate data collection, processing, analysis, and interpretation (Gui et al., 2021). Nevertheless, despite the advantages it offers, this rapid adaptation has raised ethical concerns because of the various risks it entails (Igbokwe, 2024), leading many studies to examine the challenges and opportunities it presents in the field of school leadership and management (Ghamrawi et al., 2024; Hou et al., 2019; Karakose, 2024; Wang, 2021b).

The rapid advancement of AI and GenAI has also raised concerns about the extent to which school leaders are prepared for these technologies, prompting a considerable body of research to focus on digital readiness and digital transformation (Adams and Thompson, 2025; Anysiadou and Gkliati, 2025; Kilinç et al., 2025). The incorporation of questions about the self-efficacy of school leaders and teachers in using AI (Bellibaş et al., 2025; Berkovich and Eyal, 2025) has further broadened the scope of studies on AI in school leadership and management. Developments in this field have made it possible to examine the use of AI in educational leadership across a wide spectrum, ranging from curriculum development to more granular applications such as virtual reality and augmented reality (Polat et al., 2025).

Studies on the integration of AI applications into the field of school management and leadership (e.g., Arar et al., 2026; Berkovich, 2025; Kafa, 2025a; Richardson et al., 2025) have, on the one hand, sought to develop thematic clusters and taxonomies in order to understand how the intellectual foundations of research across different areas have evolved (Sposato, 2025; Tyson and Sauers, 2021), while other studies have attempted to re-veal how AI adoption in schools is perceived from the perspectives of school leaders (Bixler and Ceballos, 2025; Marrone et al., 2025).

Taken together, these efforts indicate that research on AI in the field of school leadership and management has been steadily expanding, while also producing a fragmented body of knowledge shaped by divergent thematic and conceptual orientations. Existing literature reviews have made important contributions to understanding the effects of AI on school leadership and management, ethical issues, leaders' readiness levels, and adoption processes (Berkovich, 2025; Ghamrawi et al., 2024; Kafa, 2025a). However, these studies are largely based on narrative or limited bibliometric approaches and are therefore unable to account simultaneously for how the latent thematic structure of the field is formed, the extent to which these themes are coherent and distinct from one another, which themes have reached conceptual maturity, and whether the semantic network formed by core concepts is fragmented or integrated. Therefore, the literature on AI and school leadership and management requires a multilayered analytical framework that examines not only “what themes exist,” but also how these themes are structured, how far they have developed, and how they relate to one another.

To address this gap, the present study examines the literature on AI and school leadership and management using data mining, text mining, and a computational literature review. Accordingly, the study employs three complementary analyses. First, the latent thematic structure of the literature is identified through structural topic modeling (STM). Second, conceptual maturity analysis is used to examine whether the themes constitute emerging, developing, or mature lines of research. Finally, semantic network fragmentation analysis is employed to assess whether the structure of the field's core concepts is integrated or fragmented. In this way, unlike previous literature reviews, the present study offers a holistic analytical framework that explains the field of AI in school leadership and management by jointly considering thematic structure, topic quality, conceptual maturity, and semantic integration. This framework is significant because it has the potential to guide future research on AI in school leadership and management.

### 1.1 Theoretical background: AI leadership as a thematic, developmental, and relational knowledge domain

The field of artificial intelligence and leadership has a history dating back to the 1950s. In one of the earliest definitions in the field, McCarthy defined AI in 1955 as machines capable of imitating human cognition and making decisions (McCarthy et al., 2006). Following McCarthy, many prominent scholars, including Alan Turing, Marvin Minsky, Allen Newell, and Herbert Simon, conducted research on AI (Polat et al., 2025). The earliest traces of AI developments in school leadership and management appeared in the works of Halff (1986) and Nelson (1989); however, in these studies, leadership and management remained overshadowed by instructional technologies. After 2018, concepts such as machine learning, big data, and algorithmic analytical capacity have positioned AI as a central area of inquiry in the field of school leadership and management (Arar et al., 2026; Fullan et al., 2024; Karakose and Tulubas, 2025).

It can be argued that the first point of intersection between artificial intelligence and school leadership and management was the decision-making process. In this context, Wang (2021a) emphasized that decision-making is among the most strategic responsibilities of educational leaders and noted that AI serves as an extended brain, helping leaders make data- and evidence-based decisions. Algorithms can analyze vast amounts of data, such as examination results, demographic information, and attendance rates, far more rapidly than the human brain, thereby providing leaders with real-time insights. According to Wang, AI has the potential to support school leaders and administrators in areas such as preventing student dropout, teacher recruitment, teacher evaluation, leadership training, and even school safety. On the other hand, Wang (2021b) also pointed out that AI entails certain risks. For example, existing racial or gender-based biases in educational data may mislead administrators when embedded in teacher recruitment algorithms. Wang further noted that as schools become large repositories of data, they may also become targets of cyberattacks and student privacy violations.

Despite the risks it entails, AI has continued to occupy a central place in the field of school leadership and management because of the opportunities it offers, thereby reshaping modern school leadership as a practice that extends beyond traditional roles and is increasingly integrated with technological literacy. Adams and Thompson (2025) stated that AI can relieve administrators of the burden of routine, time-consuming tasks such as management, budgeting, scheduling, resource allocation, and data analysis, and that modernizing workflows may leave school administrators with more time for instructional leadership. Fullan et al. (2024) similarly argued that transferring routine and mechanical tasks to AI could reduce administrative burdens, thereby simplifying and modernizing school administrators' communication with students and teachers. In doing so, they maintained that leadership would remain an art in the enactment of practices requiring compassion, social intelligence, and moral responsibility.

Bixler and Ceballos (2025) likewise emphasized that school principals should not be replaced by artificial intelligence; rather, administrators should take the lead in guiding AI use. Accordingly, Bixler and Ceballos stated that school leaders and administrators could establish a symbiotic and instrumental relationship with AI by following the steps of data analysis, reviewing analytical outputs, self-directed learning, examining alternative courses of action, and developing an action plan. Bixler and Ceballos noted in particular that AI is an effective tool for identifying school- and person-specific solutions through self-directed learning. Drawing on articles on artificial intelligence, Richardson et al. (2025) also stated that administrative processes such as screening job applications, organizing data, using data to improve school effectiveness, and predicting staff turnover could be delegated to AI, thereby enabling administrators to manage their time more effectively. Richardson et al. highlighted the importance of reconfiguring education systems around AI rather than merely integrating AI into existing systems; nevertheless, they emphasized that administrators should combine their contextual knowledge and social intelligence with the analytical power of AI.

The potential contributions of AI to administrative processes in school leadership and management, particularly its facilitative role in communication and its benefits for time management, have led to a substantial increase in the number of studies on AI in this field within a relatively short period. Accordingly, studies employing systematic review and bibliometric analysis have begun to examine the theoretical framework and thematic clusters of research on AI in the field of school leadership and management. In one of the earliest examples of such reviews, Tyson and Sauers (2021) grouped the clusters they identified under the headings of leadership behaviors and interpersonal skills. Based on these findings, Tyson and Sauers suggested that school administrators should be individuals who embrace technology and possess curiosity and a commitment to lifelong learning. They further stated that AI leaders should align with the broader goals of their school district and could draw on their AI-related skills to maintain focus on all stakeholders. In the taxonomy developed by Sposato (2025), the identified subclusters comprised AI for administrative efficiency; AI for personalized learning; AI for enhancing instructional practices; AI in decision-making and policy formulation; AI for improving student support services; AI in organizational leadership and strategic planning; AI for governance and compliance; AI for community engagement and communication; ethical AI leadership and governance; and AI for diversity, equity, and inclusion initiatives. The considerable diversification of these subclusters over the approximately 4-year period between the two studies indicates that AI has moved beyond administrative processes and time management, extending into various other domains of leadership and management.

Indeed, the keyword co-occurrence analyses conducted by Arar et al. (2026) yielded clusters related to leadership concepts and decision-making; AI models and optimization; the future of work and automation; and perceptions and mediating roles. In the same study, trust perception, leadership performance, choice exploration, and big data were identified as motor themes. Basic themes were reported as AI management impact, care risk intervention, internet safety, and design behavior dynamics, whereas niche themes were identified as bias, search algorithms, research productivity, and cognitive impairment. Themes such as attitudes, adoption, classification, age, and language were characterized as either emerging or declining themes. In related studies, Ghamrawi et al. (2024) identified competency clusters such as technological literacy, adaptability, coaching, data-driven decision-making, and human-centeredness, whereas Karakose et al. (2023) pointed to subdimensions including digital leadership, technology integration, school culture, professional development, data-informed decision-making, and digital citizenship.

Polat et al. (2025), in contrast, organized the thematic clusters into challenge and benefit clusters. Within this classification, personalized learning experiences, data analytics and decision-support systems, the automation of administrative processes, and the enhancement of pedagogical quality are positioned within the benefit cluster, while technological infrastructure and resources, lack of training and awareness, data privacy and security, and ethical considerations are placed within the challenge cluster.

In sum, although existing literature reviews and bibliometric analyses in the field converge on certain common points, AI research in school leadership and management has yet to provide a sufficiently clear account of the theoretical framework, intellectual underpinnings, and patterns of knowledge production in this area. Numerous taxonomy-building studies, bibliometric analyses, and systematic literature reviews have identified distinct thematic clusters, suggesting that AI research in school leadership and management is fragmented. The present study is significant in that it identifies the intellectual foundations and semantic relationships underpinning AI research in school leadership and management and descriptively assesses the relative maturity levels of its themes. This theoretical positioning also informs the methodological design of the study. If AI research in school leadership and management is an emerging and potentially fragmented knowledge domain, then it is necessary to examine not only which themes are present, but also how these themes are structured, how conceptually consolidated they have become, and how the core concepts of the field are related to one another. For this reason, structural topic modeling is used to identify the latent thematic organization of the literature, conceptual maturity analysis is used to evaluate the developmental status and consolidation of these themes, and semantic network fragmentation analysis is used to determine whether the field's core concepts form an integrated or fragmented semantic structure. In this way, the theoretical assumption that AI leadership research is thematically diverse and conceptually unsettled is directly connected to the analytical strategy adopted in the study.

## 2 Materials and methods

### 2.1 Research design

This study was designed as a computational literature review using text mining to examine the thematic, conceptual, and semantic structure of the AI literature on school leadership and management. Structural topic modeling (STM), conceptual maturity analysis, and semantic network fragmentation analysis were used in combination to uncover latent patterns in large-scale textual data. STM was selected because it enables the identification of latent thematic structures and permits document-level metadata to be incorporated into the model. In the present study, this feature was used primarily to examine topic prevalence and temporal patterns in the literature (Roberts et al., 2019; Mishra et al., 2026). In addition, semantic coherence and exclusivity measures were used to assess whether the topics obtained were both interpretable and distinguishable from one another (Mimno et al., 2011; Roberts et al., 2014). Finally, semantic network analysis based on a co-word approach was applied to examine whether the connections among the core concepts in the field formed an integrated or fragmented structure (Callon et al., 1991; He, 1999).

The choice of these three methods was guided by the theoretical framing of AI leadership as an emerging, multi-dimensional, and relational knowledge domain. STM was used to identify the latent thematic axes of this domain; conceptual maturity analysis was used to evaluate the extent to which these axes had become stable and differentiated lines of inquiry; and semantic network fragmentation analysis was used to assess whether the field's core concepts were organized as an integrated knowledge structure or as disconnected conceptual clusters.

### 2.2 Corpus construction

The research corpus was constructed through a two-stage process of data searching, screening, and extraction, following the PRISMA 2020 guidelines. In the first data search cycle, the Web of Science (WoS) and Scopus databases were searched, with the titles, abstracts, and keywords of the studies taken as the basis for retrieval. Full-text searching was not conducted because such searches risk including irrelevant records in the dataset when the target concepts appear only in references, footnotes, or secondary contexts (Kumar and Srivastava, 2022; Mishra et al., 2026). The search string was structured to combine artificial intelligence terms with school leadership and management terms:

(“Artificial Intelligence” OR “AI”) AND (“school leader^*^” OR “school leadership” OR “principal^*^” OR “school principal^*^” OR “school administrator^*^” OR “school administration” OR “educational administration” OR “school manager^*^” OR “school management” OR “educational manager^*^” OR “educational management” OR “educational leader^*^” OR “educational leadership” OR “headteacher^*^” OR “head teacher^*^” OR “headship^*^” OR “vice-principal^*^” OR “vice principal^*^” OR “assistant principal^*^” OR “deputy principal^*^”* OR “deputy head teacher” OR “deputy headmaster”*).

The initial corpus was constructed before April 2026. To improve transparency and reproducibility, the search strategy was subsequently checked through a verification search conducted on 6 July 2026. In WoSCC, the search was conducted in the Topic field, which includes title, abstract, author keywords, and Keywords Plus. In Scopus, the same conceptual search was conducted using the TITLE-ABS-KEY field. The search was limited to studies published between 2021 and 2026. Only English-language scholarly publications were included. Articles, early access articles, reviews, and conceptual papers were considered eligible when they explicitly addressed artificial intelligence or generative AI in relation to school leadership, school management, educational administration, principal leadership, teacher leadership, governance, or educational decision-making. Records were excluded when they focused solely on higher education, generic educational technology, classroom learning without a leadership or management connection, technical AI model development without educational leadership relevance, or AI in non-educational organizational contexts.

In the first data search cycle, a total of 260 records were retrieved, comprising 86 records from WoSCC and 174 from Scopus. After 81 duplicate records between WoSCC and Scopus had been removed, 179 records were screened. At this stage, 115 records were excluded for various reasons, and 64 records from the first cycle were transferred to the corpus. In the second data search cycle, ERIC and Google Scholar were used as supplementary sources to reduce the risk of database-specific coverage bias and to identify relevant studies that may not have been indexed in WoSCC or Scopus. This second cycle did not apply broader or more flexible inclusion criteria; rather, it used the same conceptual scope and eligibility rules as the first cycle. Because ERIC and Google Scholar do not support identical Boolean search functions in the same way as WoSCC and Scopus, the original search string was adapted into shorter query combinations while retaining the two core conceptual blocks: artificial intelligence terms and school leadership/management terms. The supplementary query combinations used in ERIC and Google Scholar included the following combinations: “artificial intelligence” AND “school leadership”; “artificial intelligence” AND “school management”; “artificial intelligence” AND “educational administration”; “AI” AND “school principal”; “generative AI” AND “school leadership”; “ChatGPT” AND “school leadership”; “AI” AND “educational leadership”; and “AI” AND “teacher leadership.” These shorter query combinations were used to preserve the conceptual equivalence of the WoSCC and Scopus search strategy while adapting it to the technical limits of ERIC and Google Scholar.

For Google Scholar, the supplementary verification search was conducted on 6 July 2026. Because Google Scholar does not allow the same level of reproducible field restriction as WoSCC or Scopus, the search was conducted using the shorter query combinations listed above. For each query combination, the first 100 results, corresponding to the first 10 Google Scholar results pages, were screened in relevance order. Records were considered for further screening only when the title, abstract, snippet, or available bibliographic information indicated a direct connection between artificial intelligence and school-level leadership, management, administration, governance, teacher leadership, or educational decision-making. Google Scholar was therefore used as a supplementary coverage check rather than as an independent source with broader eligibility criteria.

**Figure 1:**
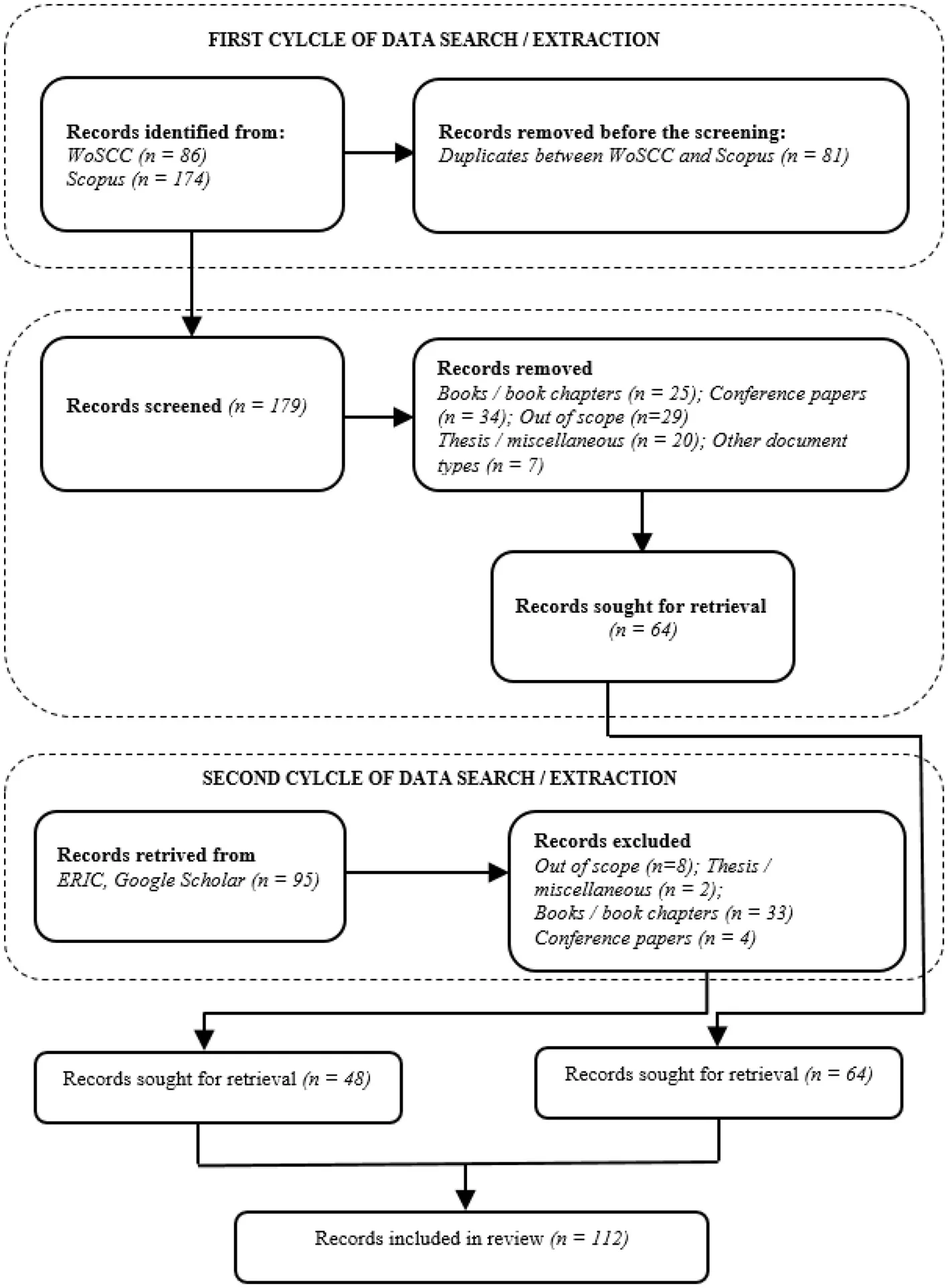
PRISMA 2020 flow diagram for the corpus construction.

The same inclusion and exclusion criteria were applied across both search cycles. Studies were included if they focused on artificial intelligence or generative AI in relation to school-level leadership, management, administration, governance, decision-making, teacher leadership, or leadership capacity. Studies were excluded if they focused solely on higher education, generic educational technology, classroom learning without a leadership or management connection, technical AI model development without educational leadership relevance, or AI in non-educational organizational contexts. Duplicate records were identified through DOI, title, author names, and publication year. For the supplementary search cycle, each potentially relevant record was logged with its source, title, authors, year, publication outlet, DOI or URL where available, screening decision, and reason for exclusion where applicable. The verification search was used to clarify and document the supplementary search procedure; it did not change the eligibility criteria or the reported corpus size. Consequently, the final research corpus consisted of 112 articles. The data searching, screening, and extraction process is presented in the PRISMA 2020 flow diagram in Figure 1.

### 2.3 Text pre-processing

Prior to the STM analysis, a single text field was created for each article by combining the title, abstract, keywords, and, where accessible, the conclusion/discussion section. Because the text corpus in the STM package consists of a document list, a vocabulary, and document-level metadata, particular care was taken to ensure that the text field was matched at the same record level with the publication year variable used in the temporal topic-prevalence analysis (Roberts et al., 2019). Other document-level variables, such as region, country context, publication type, research design, and index information, were used only for descriptive corpus profiling and were not modeled as STM prevalence covariates (Marginson, 2022).

This procedure ensured consistency in the types of textual material used across documents. Although the length of abstracts, keywords, and conclusion/discussion sections naturally varied across articles, no article in the final STM corpus was represented only by title, abstract, and keywords while others were represented by additional conclusion/discussion text. Therefore, the potential bias associated with unequal text-field composition was minimized at the corpus construction stage.

The texts were standardized prior to analysis. As part of this process, all text was converted to lowercase, and punctuation marks, numbers, excess spaces, non-alphanumeric characters, and general stop words were removed. In addition, to ensure that topic modeling focused on the conceptual structure of the corpus, words that frequently occur in academic texts but have limited power for thematic differentiation, such as “study,” “research,” “article,” “findings,” “result,” “method,” and “purpose,” were removed from the dataset (Kumar and Srivastava, 2022; Roberts et al., 2019). At the final stage, certain terms specific to artificial intelligence and school leadership/management were retained. In particular, multi-word expressions such as “artificial intelligence,” “generative AI,” “ChatGPT,” “school leadership,” “educational leadership,” “school management,” “teacher leadership,” and “instructional leadership” were treated as compound terms prior to analysis to prevent loss of meaning. Stemming was not applied, as singular, plural, or derived word forms may carry different contextual meanings in certain cases. This decision is consistent with previous STM applications, indicating that reducing words to their stems may weaken some conceptual distinctions (Kumar and Srivastava, 2022).

### 2.4 Structural topic modeling

In the first stage of analysis, structural topic modeling was used to identify the latent thematic structure of the AI literature in school leadership and management. STM is a probabilistic topic model that treats each document as a mixture of multiple topics. In this respect, STM follows the logic of Latent Dirichlet Allocation (LDA), yet unlike LDA, it can incorporate document-level metadata into the model. In the present study, publication year was the only metadata variable used to examine temporal variation in topic prevalence. Other document-level variables were retained for descriptive corpus characterization and were not used as STM prevalence or content covariates (Kuhn, 2018; Roberts et al., 2014, 2019).

In the STM model, each article was defined as a document. For each document, the analysis text was created by combining the title, abstract, keywords, and, where accessible, the conclusion/discussion section. In the model, the topic distribution of each document was linked to document-level metadata. Within this framework, the document–topic distribution is expressed by the following logistic-normal model:


$$\begin{array}{l} \theta_{d}|X_{d}\gamma ,\Sigma \sim \text{LogisticNormal}\left(\mu =X_{d}\gamma ,\Sigma \right)\; \end{array}$$

Here, θ_*d*_ denotes the distribution of topics in document *d*; *X*_*d*_ denotes the document-level metadata vector, which in the present analysis included publication year for modeling temporal variation in topic prevalence; γ denotes the metadata coefficients; and Σ denotes the covariance matrix among topics. This structure allows topics to be modeled not as fixed distributions across documents but as probabilistic distributions that may vary with document characteristics (Roberts et al., 2019).

In STM, topic content is also modeled through word distributions. The word distribution associated with a given topic is estimated as a function of baseline word frequency, topic-specific deviations, and, where applicable, content covariates:


$$\begin{array}{l} \beta_{d,k}\propto \exp\left(m+\kappa_{k}^{\left(t\right)}+\kappa_{y_{d}}^{\left(c\right)}+\kappa_{y_{d},k}^{\left(i\right)}\right) \end{array}$$

In this equation, β__*d*_, *k*_ denotes the word distribution for topic *k* in document *d*; *m* denotes the baseline word frequency; $\kappa_{k}^{\left(\text{t}\right)}$ denotes the topic-specific deviation; $\kappa_{yd}^{\left(\text{c}\right)}$ denotes the deviation associated with the content covariate; and $\kappa_{yd,k}^{\left(i\right)}$ denotes the interaction between the topic and the content covariate. In this way, the model estimates not only which topics are present, but also the word patterns through which these topics are constituted (Kuhn, 2018; Roberts et al., 2019).

The words in each document were modeled through a two-stage generative process. First, a topic is assigned to each word from the document-specific topic distribution:


$$\begin{array}{l} z_{d,n}|\theta_{d}\sim \text{Multinomial}\left(\theta_{d}\right) \end{array}$$

The observed word is then generated conditional on the selected topic:


$$\begin{array}{l} w_{d,n}|z_{d,n},\beta_{d,k=z_{d,n}}\sim \text{Multinomial}\left(\beta_{d,k=z_{d,n}}\right) \end{array}$$

Here, *z*_*d, n*_ denotes the topic assignment of the nth word in document d, while *W*_*d, n*_ denotes the observed word. The model parameters were estimated using the partially collapsed variational expectation–maximization algorithm implemented in the stm package. This approach approximates posterior distributions that are difficult to compute directly and, upon convergence, produces document–topic proportions and topic–word distributions (Roberts et al., 2014, 2019).

Alternative STM models were estimated for different numbers of topics and compared on semantic coherence, exclusivity, held-out likelihood, residual values, and interpretability. Semantic coherence indicates the extent to which the most probable words within a topic co-occur in the same documents, whereas exclusivity makes it possible to assess the degree to which these words are distinguishable from those associated with other topics. Model selection therefore took into account not only statistical adequacy, but also whether the model produced a structure that was internally meaningful and distinguishable across topics. The resulting topics were labeled by jointly examining FREX words, the highest-probability words, and the representative articles most strongly associated with each topic. This process ensured that the STM outputs were evaluated not merely as automated model results, but in conjunction with researcher interpretation and domain knowledge. Model selection was not based on a single statistical criterion; instead, semantic coherence, exclusivity, held-out likelihood, residual values, and interpretability were evaluated together. Because there is no single correct number of topics in topic modeling, it is recommended that the final decision be made by using statistical diagnostic measures together with domain knowledge (Kuhn, 2018; Lindstedt, 2019; Roberts et al., 2014, 2019).

### 2.5 Conceptual maturity analysis

Conceptual maturity analysis was used to assess not only the visibility of topics identified in the AI literature on school leadership and management, but also the extent to which they have developed into coherent, established lines of inquiry. In the maturity model literature, maturity is conceptualized as a framework that evaluates the developmental level of a field, process, or competency in a staged manner. Such models typically describe the development of specific objects or processes through sequential levels and enable assessment of their current state (Sadiq et al., 2021; Tarhan et al., 2016; Wendler, 2012).

In this study, conceptual maturity was calculated for each topic as a descriptive composite index based on five normalized indicators: overall topic prevalence, peak topic visibility, temporal continuity, semantic coherence, and exclusivity. Topic prevalence indicates the overall weight of a given topic within the corpus; peak topic visibility captures the highest annual visibility reached by the topic; temporal continuity indicates whether the topic maintains visibility across the observed publication years; semantic coherence reflects whether the words within the topic form a meaningful whole; and exclusivity indicates the extent to which the topic is differentiated from other topics. The CMI was not intended as an inferential or predictive statistic, but as a comparative descriptive tool for assessing the relative consolidation of the themes identified by STM.

The conceptual maturity score was calculated as follows:


$$\begin{array}{l} CMI_{k}=\frac{P_{k}^{*}+PV_{k}^{\;*}+TC_{k}^{\;*}+C_{k}^{\;*}+E_{k}^{\;*}}{5}\times 100 \end{array}$$

Here, *CMI*_*k*_ denotes the conceptual maturity index of topic *k*; $P_{k}^{\;*}$ denotes normalized overall topic prevalence; $PV_{k}^{\;*}$ denotes normalized peak topic visibility; $TC_{k}^{\;*}$ denotes normalized temporal continuity; $C_{k}^{\;*}$ denotes normalized semantic coherence; and $E_{k}^{\;*}$ denotes normalized exclusivity (Wendler, 2012).

The five indicators were weighted equally because there is no established theoretical or empirical basis in the STM or maturity-model literature for privileging one dimension of conceptual maturity over the others in this specific research context. Equal weighting was therefore adopted as a transparent baseline strategy. This choice assumes that conceptual maturity is jointly reflected in visibility, temporal persistence, internal semantic coherence, and conceptual distinctiveness. To avoid overinterpreting the index, CMI scores are interpreted only as broad comparative indicators of relative maturity rather than as precise measurements of the developmental status of each topic. Because the variables were generated on different scales, each component was transformed to a range between 0 and 1 using min–max normalization:


$$\begin{array}{l} X_{k}^{\;*}=\frac{X_{k}-\min\left(X\right)}{\max\left(X\right)-\min\left(X\right)} \end{array}$$

In this formula, $X_{k}^{*}$ denotes the normalized value of the relevant component; *X*_*k*_ denotes the raw value for topic *k*; and min(*X*) and max(*X*) denote the lowest and highest values of the same component across all topics, respectively (Wendler, 2012). Because min–max normalization was performed across only the five topics identified in this corpus, the normalized values should be interpreted as relative scores within the present topic solution. A value of 0.00 does not indicate the absence of prevalence, continuity, coherence, or exclusivity; rather, it indicates that the topic had the lowest value among the five topics on that specific indicator. Similarly, a value of 1.00 indicates the highest relative value within this corpus, not an absolute maximum level of maturity.

Because the corpus covers a short and uneven time span, and because the number of studies in the earliest years is small, the temporal component was interpreted cautiously as an indicator of temporal continuity rather than as a precise estimate of linear growth or decline. Annual topic distributions were used descriptively to assess whether a topic appeared as a sustained line of inquiry across the observed period or whether it was concentrated in a limited time window. Accordingly, the temporal component should not be read as a statistically stable trend coefficient; it indicates only the relative continuity of topic visibility within the corpus.

The obtained CMI_k_ values were interpreted at three levels. For descriptive classification, CMI scores below 40 were categorized as low maturity, scores from 40 to 69 as moderate maturity, and scores of 70 or above as high maturity. These thresholds were used as heuristic interpretive boundaries for comparative purposes within the present corpus and should not be understood as externally validated or inferential cut-off points. Low conceptual maturity refers to topics with limited prevalence, temporal instability, and weak semantic differentiation. Moderate conceptual maturity was applied to topics that had achieved some visibility but had not yet developed a strong conceptual core. High conceptual maturity, in turn, refers to topics characterized by high prevalence, a stable or increasing temporal trend, strong semantic coherence, and clear exclusivity (Wendler, 2012).

The purpose of this analysis was not to propose a normative optimal level, but to descriptively compare the developmental status of the themes within the literature. For this reason, conceptual maturity analysis was used not as a prescriptive model, but as a descriptive evaluative tool that indicates which themes in the field have become established and which remain at an early or fragmented stage of development. This approach is consistent with the maturity model literature, which suggests that maturity models can be used as descriptive tools for assessing existing capacity or developmental level (Wendler, 2012; Sadiq et al., 2021).

### 2.6 Semantic network fragmentation analysis

Semantic network fragmentation analysis was used to examine the extent to which core concepts in the AI literature on school leadership and management are connected to one another. This analysis is based on the co-word approach. Co-word analysis evaluates the relationships among concepts within a research field through patterns of their co-occurrence in the same documents. For this reason, the method is well-suited to examining whether the field's conceptual structure is integrated or fragmented (Callon et al., 1991; He, 1999).

In the analysis, a term dictionary was first prepared, comprising core concepts such as artificial intelligence, school leadership, ethics, decision-making, digital leadership, teacher leadership, management, policy, human-centered artificial intelligence, and pedagogical transformation. This dictionary-based strategy was adopted deliberately because the purpose of the semantic network analysis was not to extract all possible terms in the corpus inductively, but to examine the relational structure among theoretically and empirically central concepts in the field. Dictionary-based text analysis is widely used to operationalize predefined constructs in textual data, and co-word analysis is commonly used to map conceptual associations among terms through their co-occurrence in documents. Therefore, the controlled dictionary was used as an analytical boundary that made the integration/fragmentation analysis transparent, replicable, and aligned with the study's research questions. Subsequently, the presence or absence of these concepts in each article was coded, and a co-occurrence matrix among the terms was constructed. The co-occurrence of two concepts within the same article was treated as a co-word-based semantic association between those concepts, rather than as direct evidence of a causal or fully theorized relationship. Because “artificial intelligence” was the search-anchor concept of the corpus and appeared across the included records, it was retained in the controlled dictionary for transparency but excluded from the network fragmentation metrics and normalized co-occurrence checks. This exclusion was made to avoid mechanically inflating network connectivity through a term that defined the corpus itself. The semantic network analyses therefore focused on 23 analytically informative concepts. The fully controlled dictionary, including standardized concept labels, broader conceptual categories, synonym/variant expressions, and coding rules, is provided in Appendix A.

The co-occurrence value between terms was calculated as follows:


$$\begin{array}{l} C_{ij}=\overset{N}{\underset{d=1}{\sum }}x_{id}x_{jd} \end{array}$$

Here, *C*_*ij*_ denotes the number of co-occurrences between terms *i* and *j*; *x*_*id*_ indicates whether term *i* appears in document *d*; *x*_*jd*_ indicates whether term *j* appears in the same document; and *N* denotes the total number of documents. If the relevant term appeared in the document, it was coded as 1; if not, it was coded as 0 (Callon et al., 1991; He, 1999). Because article-level co-occurrence is a broad unit of analysis and may overstate conceptual connectivity when frequently occurring concepts appear across many documents, a Jaccard-normalized co-occurrence check was also conducted. Pairwise Jaccard similarity was calculated for each concept pair as follows:


$$\begin{array}{l} J_{ij}=C_{ij}/\left(f_{i}+f_{j}-C_{ij}\right) \end{array}$$

Here, *C*_*ij*_ denotes the number of articles in which concepts i and j co-occur; *f*_*i*_ denotes the number of articles containing concept *i*; and *f*_*j*_ denotes the number of articles containing concept j. Unlike raw co-occurrence counts, Jaccard similarity normalizes the pairwise association by the total number of documents in which either concept appears. This check was used to assess whether the integrated structure observed in the raw co-occurrence network was driven primarily by high-frequency general concepts.

Network density was calculated to indicate the overall level of connectivity among concepts:


$$\begin{array}{l} D=\frac{2L}{n\left(n-1\right)} \end{array}$$

Here, *D* denotes network density; *L* denotes the number of observed links; and n denotes the total number of terms in the network. High density indicates that concepts are more frequently connected to one another, whereas low density suggests a sparser and more fragmented conceptual structure (Callon et al., 1991; He, 1999).

The degree of network fragmentation was assessed using the network's component structure. The proportion of the largest component within the network and the presence of disconnected or weakly connected subclusters were taken into account:


$$\begin{array}{l} FI=1-\frac{LC}{n} \end{array}$$

Here, *FI* denotes the fragmentation index; *LC* denotes the number of terms in the largest component; and *n* denotes the total number of terms in the network. An *FI* value approaching 0 indicates that the concepts are largely concentrated within a single integrated network, whereas an *FI* value approaching 1 indicates a more fragmented conceptual structure (Callon et al., 1991; He, 1999).

In addition, the modularity value of the network was calculated to examine whether the concepts formed distinct subclusters or thematic communities:


$$\begin{array}{l} Q=\frac{1}{2m}\underset{i,j}{\sum }\left[A_{ij}-\frac{k_{i}k_{j}}{2m}\right]\delta \left(c_{i},c_{j}\right) \end{array}$$

Here, *Q* denotes the modularity value; *A*_*ij*_ denotes the link between terms *i* and *j*; *k*_*i*_ and *k*_*j*_ denote the degree values of the respective terms; *m* denotes the total number of links; and δ(*c*_*i*_,*c*_*j*_) denotes the function indicating whether the two terms belong to the same community. High modularity indicates that the field is organized around conceptually distinct clusters (Callon et al., 1991; He, 1999).

The purpose of this analysis was not merely to list the key concepts in the field but to reveal the relational structure among them. In this way, the latent thematic structure identified by STM was complemented by co-word-based semantic network analysis, enabling an assessment of whether the field has developed in a conceptually integrated or fragmented manner.

### 2.7 Validity, reliability, and interpretation process

In this study, the outputs from the computational analyses were not treated as final findings; rather, they were evaluated alongside the researcher's interpretation, domain knowledge, and document-level checks. Because topic modeling approaches are based on unsupervised learning, there is no predefined “correct” topic structure. For this reason, statistical criteria and researcher judgment were used together in the processes of topic labeling, model selection, and conceptual interpretation. This approach is consistent with studies emphasizing that automated text analysis does not replace human interpretation; rather, it supports the researcher's interpretive process by making patterns within large text corpora visible (Grimmer and Stewart, 2013; Lindstedt, 2019; Roberts et al., 2014).

The determination of topic labels was not based solely on the most frequent words. For each topic, FREX words, highest-probability words, and the representative articles most strongly associated with the relevant topic were examined together. This method is consistent with previous studies indicating that word clusters and representative documents should be evaluated together when interpreting STM outputs (Kumar and Srivastava, 2022; Mishra et al., 2026).

Checklists were used during the stages of making inclusion and exclusion decisions, removing duplicate records, evaluating borderline studies, and assigning topic labels. Borderline studies were further reviewed for relevance to the research scope; only those that established a clear connection between artificial intelligence and school leadership, school management, educational administration, teacher leadership, or educational decision-making processes were retained in the final corpus. In this way, both the dataset's scope consistency and analytical tractability were preserved.

To further reduce the risk of selection bias, the supplementary records identified through ERIC and Google Scholar were not treated as automatically eligible. They were subjected to the same inclusion and exclusion criteria as the records retrieved from WoSCC and Scopus. A screening checklist was used to document the source of each record, the basis for its relevance, and the reason for exclusion where applicable. This procedure was intended to make the supplementary search transparent, auditable, and consistent with the scope of the study.

## 3 Results

**RQ1**. *What latent thematic patterns structure the emerging literature on artificial intelligence in school leadership and management, and how do these themes vary over time?*

To examine the latent thematic structure of the AI literature on school leadership and management and to identify the dominant thematic clusters and their temporal patterns, a corpus comprising 112 studies was analyzed.

As shown in Table 1, the corpus contains the largest number of studies from 2025. Because the corpus includes records available up to the stated search cut-off and verification procedure, the 2026 count should be interpreted as representing a partial publication year rather than the full annual output for 2026. Based on the number of studies identified in the field of AI in school leadership and management, the region with the highest number of published articles is the Middle East and Africa. This region is followed by studies conducted globally or based on data collected across multiple countries. Empirical studies account for 52.68% of the studies conducted in the field. Accordingly, nearly one in two studies in the field of school leadership and management consists of theoretical work, such as literature reviews, bibliometric analyses, or editorials. Consistent with this pattern, review and synthesis studies constitute the most frequently preferred research design. Among empirical studies, quantitative and qualitative approaches are roughly evenly distributed, whereas mixed-methods studies account for only 5.36% of the corpus.

**Table 1:** Research corpus.

Category	Group	*n* (documents)	%
Year	2021	3	2.68
	2022	0	0
	2023	3	2.68
	2024	19	16.96
	2025	63	56.25
	2026	24	21.43
Region	Middle East and North Africa	28	25
	Global or mixed	26	23.21
	Asia	18	16.07
	Africa	13	11.61
	Europe	12	10.71
	North America	12	10.71
	Other or unknown	2	1.79
	Oceania	1	0.89
Document type	Empirical	59	52.68
	Review	28	25
	Conceptual	25	22.32
Research design	Review or synthesis	39	34.82
	Qualitative	29	25.89
	Quantitative	28	25.89
	Conceptual or theoretical	7	6.25
	Mixed methods	6	5.36
	Other	2	1.79

In the Structural Topic Modeling analysis, nine STM models, ranging from 4 to 12 topics, were first tested to determine the number of topics and to understand how the dominant topics in the field were grouped. The models were compared according to held-out likelihood, residual values, semantic coherence, and exclusivity. A higher held-out likelihood value, a lower residual value, a semantic coherence value closer to zero, and a higher exclusivity value were considered positive indicators in model selection.

**Figure 2:**
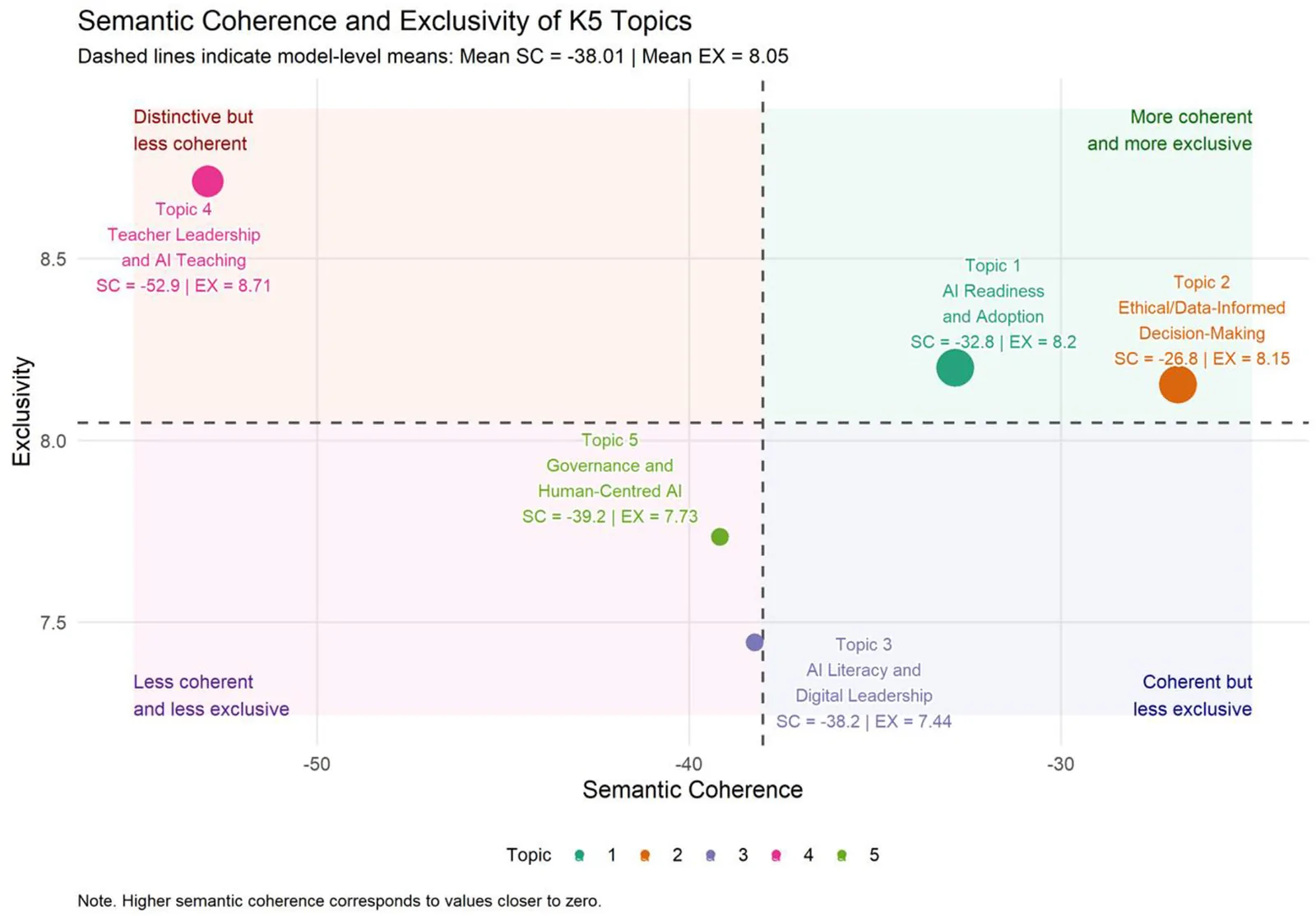
Semantic coherence and exclusivity of K5 topics.

As shown in Table 2, the *K* = 5 model produced the most balanced solution among the alternative models tested. This model had the highest held-out likelihood, the lowest residual, and the strongest semantic coherence. Although exclusivity values increased with higher *K*, this increase was accompanied by weaker semantic coherence and higher residual values. This suggests that, although the model produced some more distinctive terms as the number of topics increased, it also over-fragmented the thematic structure. For this reason, the five-topic model was selected as the final STM solution. To support the interpretability of the selected *K* = 5 model, the semantic coherence and exclusivity values of the topics in the final model were also visualized. This visualization was used not as an independent analysis, but as a topic-level diagnostic output of the STM model. As shown in Figure 2, Topic 1 and Topic 2 have both exclusivity values above the model average and semantic coherence values closer to zero. This indicates that these two topics are both more internally coherent and more clearly distinguishable from the other topics. Topic 4, by contrast, has the highest exclusivity value, yet displays a weaker profile in terms of semantic coherence. This finding suggests that the theme of teacher leadership and AI-integrated teaching is clearly differentiated from the other topics, but that it contains a more heterogeneous word pattern internally. Topic 3 and Topic 5, with values below the model average, were evaluated as broader and relatively less differentiated conceptual clusters.

**Table 2:** Structural topic modeling results.

*K*	Held-out likelihood	Residual	Semantic coherence	Exclusivity
4	−6.091	1.294	−46.769	7.926
5	−6.043	1.270	−46.310	8.124
6	−6.170	1.275	−47.614	8.322
7	−6.188	1.369	−50.062	8.414
8	−6.347	1.328	−48.796	8.388
9	−6.328	1.388	−51.933	8.464
10	−6.444	1.427	−58.338	8.598
11	−6.329	1.410	−53.484	8.548
12	−6.410	1.472	−56.591	8.671

The distribution of the estimated models according to held-out likelihood, semantic coherence, exclusivity, and residual values is presented below. The dominance rates and expected prevalence levels of the topics in the selected model are presented in the table below.

As shown in Table 3, Topic 2 has the highest expected topic proportion. This cluster, which includes studies emphasizing the use of AI for data analysis in decision-making processes in school leadership and management, as well as the need for data collection to be transparent, inclusive, and ethically compliant, is followed by the cluster composed of studies focusing on school leaders' readiness, willingness to adopt AI, and self-efficacy in using artificial intelligence. The terms contributing to the formation of the topics are presented in Table 4.

**Table 3:** The dominance rates and expected prevalence levels of the topics.

Topic ID	Topic label	Expected topic prevalence	%
2	Ethical and Data-Informed AI Decision-Making Processes In Educational Leadership	0.290	29.01
1	AI Readiness, Adoption, and Self-Efficacy in School Leadership	0.254	25.43
5	Governance Frameworks for Ethical and Human-Centered AI Leadership	0.186	18.58
4	Teacher Leadership, Distributed Agency, and Classroom Practices in AI-Integrated Teaching	0.141	14.07
3	AI Literacy, Digital Leadership, and Educational Management Capacity	0.129	12.91

**Table 4:** Topic labels and representative terms.

Topic ID	Topic label	Highest-probability terms	FREX terms	Lift terms	Score terms
1	AI Readiness, Adoption, and Self-Efficacy in School Leadership	School, leadership, study, leaders, artificial, intelligence, integration, digital, support, and use	Readiness, GenAI, generative, leaders', school, roles, ChatGPT, principals', self-efficacy, and intention	Centralized, communicate, districts, PLS-SEM, constrained, Cyprus, GenAI, readiness, and Türkiye	GenAI, intention, centralized, usefulness, perceived, Cyprus, readiness, self-efficacy, and Türkiye
2	Ethical and Data-Informed AI Decision-Making Processes in Educational Leadership	Educational, leadership, intelligence, artificial, decision-making, learning, management, education, ethical, and personalized	Personalized, data-driven, educational, privacy, decision-making, values, institutions, and transparency	Africa, careful, documents, fairness, maps, partnerships, safeguards, security, streamlining, and synthesizing	Africa, transparency, bias, privacy, evaluation, bibliometric, values, and personalized
3	AI Literacy, Digital Leadership, and Educational Management Capacity	Leadership, educational, management, education, school, artificial, intelligence, administrators, and digital	STEM, scale, smart, sustainability, quality, administrators, technologies, skills, performance, and exploratory	AI-enhanced, analyses, exploratory, gender, generic, quality, reliable, scale, smart, and structure	Quality, scale, smart, gender, STEM, sustainability, valid, state, analyses, and reliable
4	Teacher Leadership, Distributed Agency, and Classroom Practices in AI-Integrated Teaching	Leadership, teacher, study, teachers', teaching, teachers, distributed, professional, development, and innovation	Teachers', distributed, teacher, teaching, innovative, teachers, positive, beliefs, expectations, and classroom	Indirect, behavior, expectations, positive, self, beliefs, primarily, and Arab	Behavior, innovative, beliefs, positive, expectations, teachers, teachers', curriculum, effects, and self
5	Governance Frameworks for Ethical and Human-Centered AI Leadership	Leadership, school, ethical, intelligence, artificial, governance, educational, digital, and education	Governance, systematic, literature, instructional, synthesizes, human, framework, and equitable	Automate, balanced, embedding, organizational, oversight, preparation, relational, and scoping	Organizational, governance, scoping, relational, domains, and synthesizes

The final STM model showed that the artificial intelligence literature in school leadership and management is structured around five main thematic axes. The most prevalent topic is “Ethical and Data-Informed AI Decision-Making Processes in Educational Leadership,” which accounts for approximately 29.0% of the corpus. This topic is characterized by terms such as decision-making, data-driven, privacy, transparency, equity, bias, evaluation, and moral judgment. Representative studies focus on the potential of artificial intelligence to support educational leaders' data-informed decision-making, while also discussing ethical risks, including algorithmic bias, data privacy, justice, accountability, and professional judgment.

The second most prevalent topic is “AI Readiness, Adoption, and Self-Efficacy in School Leadership,” which accounts for approximately 25.4% of the corpus. This topic is characterized by terms such as readiness, GenAI, generative AI, self-efficacy, perceived usefulness, adoption, support, training, and infrastructure. Representative documents focus on school leaders' willingness to integrate generative artificial intelligence into leadership tasks, their perceived usefulness, AI literacy, perceived self-efficacy, and the conditions for organizational support.

The third topic, accounting for approximately 18.6% of the corpus, was labeled “Governance Frameworks for Ethical and Human-Centered AI Leadership.” This topic is marked by terms such as review, governance, framework, human-centered, oversight, ethics, and stakeholder. This theme mainly includes systematic reviews, scoping reviews, and conceptual framework studies. Accordingly, part of the literature appears to have focused on ethical governance, human oversight, leadership standards, and policy frameworks, rather than on the technical use of artificial intelligence itself.

The fourth topic, accounting for approximately 14.1% of the corpus, is “Teacher Leadership, Distributed Agency, and Classroom Practices in AI-Integrated Teaching.” This topic is defined by terms such as teacher, teachers, distributed, teaching, classroom, beliefs, agency, innovation, and behavior. Representative studies focus on teacher leadership in AI-supported classrooms, teacher beliefs, distributed leadership, classroom management, professional identity, and the ways in which human–AI interaction reshapes teacher authority. This finding indicates that the literature on AI leadership is not limited solely to principals or administrators but also encompasses teachers' classroom leadership practices and professional roles.

The fifth topic, accounting for approximately 12.9% of the corpus, was labeled “AI Literacy, Digital Leadership, and Educational Management Capacity.” This topic is associated with terms such as STEM, scale, administrators, skills, validation, smart, sustainability, technologies, and management systems. This theme encompasses issues such as educational administrators' AI literacy, digital leadership competencies, smart school management, educational management systems, scale development, and institutional capacity building.

Within the scope of the first analysis, the descriptive trends of the identified topics over the years were examined. The table below presents the number of articles by year, the topic proportions across years, and the corresponding percentages.

Table 5 shows that the distribution of dominant topics varies markedly across years. Although only a small number of articles were published in 2021 and 2023, the literature in this early period appears to have been concentrated primarily around the topic “Ethical and Data-Informed AI Decision-Making Processes in Educational Leadership”; in both years, two out of three articles were classified under this topic (66.67%). In 2024, topic diversity increased. Although “Ethical and Data-Informed AI Decision-Making Processes in Educational Leadership” remained the most dominant topic (42.11%), “AI Literacy, Digital Leadership, and Educational Management Capacity” also became strongly visible (26.32%). In 2025, the main focus of the literature shifted toward the topic “AI Readiness, Adoption, and Self-Efficacy in School Leadership,” which accounted for 23 of the 63 articles published that year (36.51%). In the same year, “Ethical and Data-Informed AI Decision-Making Processes in Educational Leadership” also maintained its strong position (33.33%). In 2026, however, the most notable shift was the rise of “Governance Frameworks for Ethical and Human-Centered AI Leadership”; this topic accounted for 9 of the 24 articles published that year and became the most dominant theme within the annual distribution (37.50%). Overall, the table indicates that the field was initially focused on ethical and data-informed decision-making, that discussions of AI readiness and adoption gained prominence in 2025, and that governance and human-centered AI leadership frameworks became more prominent in 2026.

**Table 5:** Descriptive trends of the identified topics over the years.

Year	Dominant topic	Topic label	Number of articles	Total articles in the same year	In year percentage (%)	General percentages of all topics (%)
2021	1	AI Readiness and Adoption	1	3	33.33	25.43
2021	2	Ethical and Data-Informed Decision-Making	2	3	66.67	29.01
2023	1	AI Readiness and Adoption	1	3	33.33	25.43
2023	2	Ethical and Data-Informed Decision-Making	2	3	66.67	29.01
2024	1	AI Readiness and Adoption	2	19	10.53	25.43
2024	2	Ethical and Data-Informed Decision-Making	8	19	42.11	29.01
2024	3	AI Literacy and Digital Leadership	5	19	26.32	12.91
2024	4	Teacher Leadership and AI-Integrated Teaching	2	19	10.53	14.07
2024	5	Governance and Human-Centered AI Leadership	2	19	10.53	18.58
2025	1	AI Readiness and Adoption	23	63	36.51	25.43
2025	2	Ethical and Data-Informed Decision-Making	21	63	33.33	29.01
2025	3	AI Literacy and Digital Leadership	2	63	3.17	12.91
2025	4	Teacher Leadership and AI-Integrated Teaching	8	63	12.70	14.07
2025	5	Governance and Human-Centered AI Leadership	9	63	14.29	18.58
2026	1	AI Readiness and Adoption	2	24	8.33	25.43
2026	2	Ethical and Data-Informed Decision-Making	4	24	16.67	29.01
2026	3	AI Literacy and Digital Leadership	5	24	20.83	12.91
2026	4	Teacher Leadership and AI-Integrated Teaching	4	24	16.67	14.07
2026	5	Governance and Human-Centered AI Leadership	9	24	37.50	18.58

**Figure 3:**
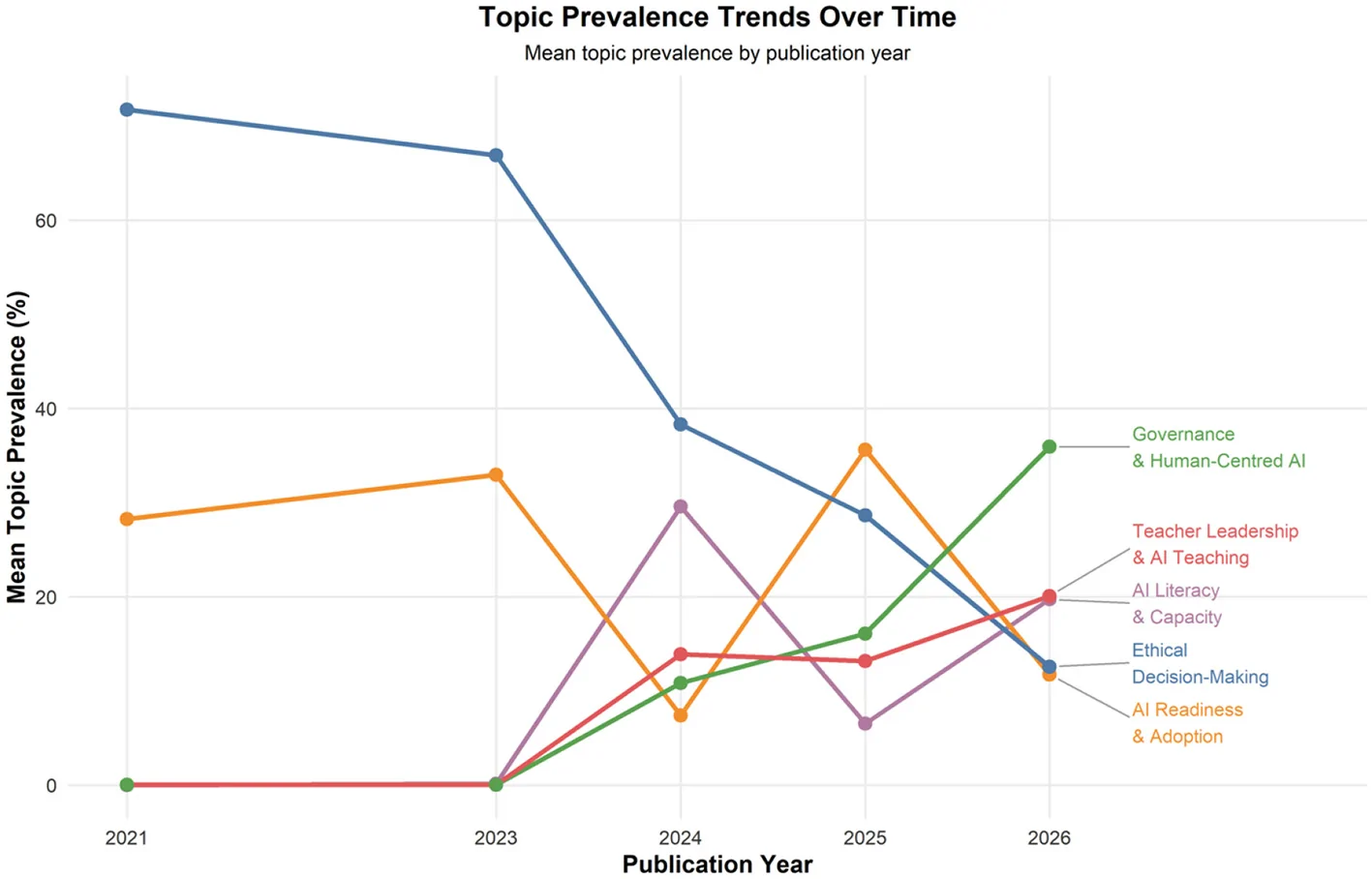
Topic prevalence trends over time.

Figure 3 suggests that over the 5-year period, the tendency to view AI merely as a tool for decision-making through data analysis has gradually declined in the field of school leadership and management. Although the acceptance of AI by school leaders and administrators, and its incorporation into practice, follows a fluctuating trajectory, the rise of the “Governance Frameworks for Ethical and Human-Centered AI Leadership” theme indicates that AI is increasingly recognized as an intelligent strategic partner in school leadership and management.

The STM findings, as a whole, indicate that the artificial intelligence literature in the field of school leadership and management has begun to crystallize around five main axes. The two most prevalent topics reveal that the field is structured primarily around AI-supported decision-making processes and school leaders' levels of AI adoption, readiness, and self-efficacy. The remaining topics point to more specialized yet theoretically significant subfields, such as ethical governance frameworks, teacher leadership in AI-integrated classrooms, and management capacity grounded in AI literacy. These results indicate that the literature treats artificial intelligence not merely as a technical tool, but as a matter of leadership, governance, ethical responsibility, and professional capacity.

**RQ2**. *To what extent has the literature on artificial intelligence in school leadership and management developed conceptually mature thematic structures, and how does this maturity vary across identified topics?*

A conceptual maturity analysis was conducted to assess the extent to which themes identified in the artificial intelligence literature on school leadership and management constitute stable, distinctive, and institutionally established conceptual structures. Whereas, the topic quality analysis conducted in the previous stage focused on semantic coherence and exclusivity values, this stage examined the maturity level of the themes through a more holistic composite index. Within this scope, the Conceptual Maturity Index (CMI) was constructed by combining five indicators: overall topic prevalence, peak topic visibility, temporal continuity, semantic coherence, and exclusivity. Each indicator was normalized and weighted equally in the calculation of the CMI. CMI values were reported on a 0–100 scale for ease of comparison across topics. Higher values indicate that a theme is more visible within the dataset, more temporally continuous, more semantically coherent, and more clearly differentiated from other themes. However, because the CMI is a descriptive composite index based on normalized indicators, these scores should not be interpreted as precise or inferential estimates. They are used to support broad maturity classifications and relative comparisons among topics.

Table 6 presents the conceptual maturity indicators calculated for the five topics and their total CMI values. The findings show that the conceptual maturity levels of the themes differ markedly from one another. Because the indicators were min–max normalized across the five-topic solution, the values in Table 6 should be read comparatively. Thus, values of 0.00 and 1.00 indicate the lowest and highest relative positions among the five topics, respectively, rather than absolute absence or full presence of a given maturity dimension.

**Table 6:** Conceptual maturity indicators.

Topic	Theme	Prevalence	Peak visibility	Temporal continuity	Semantic coherence	Exclusivity	CMI	Maturity level
2	Ethical and Data-Informed AI Decision-Making in Educational Leadership	1.000	1.000	1.000	1.000	0.559	91.2	High maturity
1	AI Readiness, Adoption, and Self-Efficacy in School Leadership	0.402	0.301	1.000	0.770	0.596	61.4	Moderate maturity
5	Governance Frameworks for Ethical and Human-Centered AI Leadership	0.092	0.307	0.000	0.528	0.229	23.1	Low maturity
4	Teacher Leadership, Distributed Agency, and AI-Integrated Teaching	0.000	0.000	0.000	0.000	1.000	20.0	Low maturity
3	AI Literacy, Digital Leadership, and Educational Management Capacity	0.051	0.184	0.000	0.563	0.000	16.0	Low maturity

As a sensitivity check, the broad maturity classifications were also examined by reducing the influence of the temporal continuity component and by considering the index without this component. These checks did not change the overall interpretation: Topic 2 remained the only high-maturity theme, Topic 1 remained at a moderate maturity level, and Topics 3, 4, and 5 remained low-maturity themes. This suggests that the maturity classifications were not driven solely by the temporal component.

According to Table 6, the highest level of conceptual maturity was observed in Topic 2, namely “Ethical and Data-Informed AI Decision-Making Processes in Educational Leadership.” This theme was classified as having a high maturity level, with a CMI score of 91.2. The fact that Topic 2 received the highest values for prevalence, peak visibility, temporal continuity, and semantic coherence indicates that this theme is both highly visible within the dataset and sustained over time. At the same time, the exclusivity value of 0.559 indicates that this theme is not fully differentiated from the other topics and shares certain conceptual elements with them.

Topic 1, namely “AI Readiness, Adoption, and Self-Efficacy in School Leadership,” demonstrates a moderate level of conceptual maturity with a CMI score of 61.4. The temporal continuity value of 1.000 for this theme is important in that it indicates visibility across all years examined. In addition, its semantic coherence value of 0.770 suggests that the theme exhibits a relatively coherent internal conceptual structure. Nevertheless, the lower prevalence and peak visibility values compared with Topic 2 suggest that, although this theme shows continuity, it has not yet fully become the field's dominant conceptual focus.

**Figure 4:**
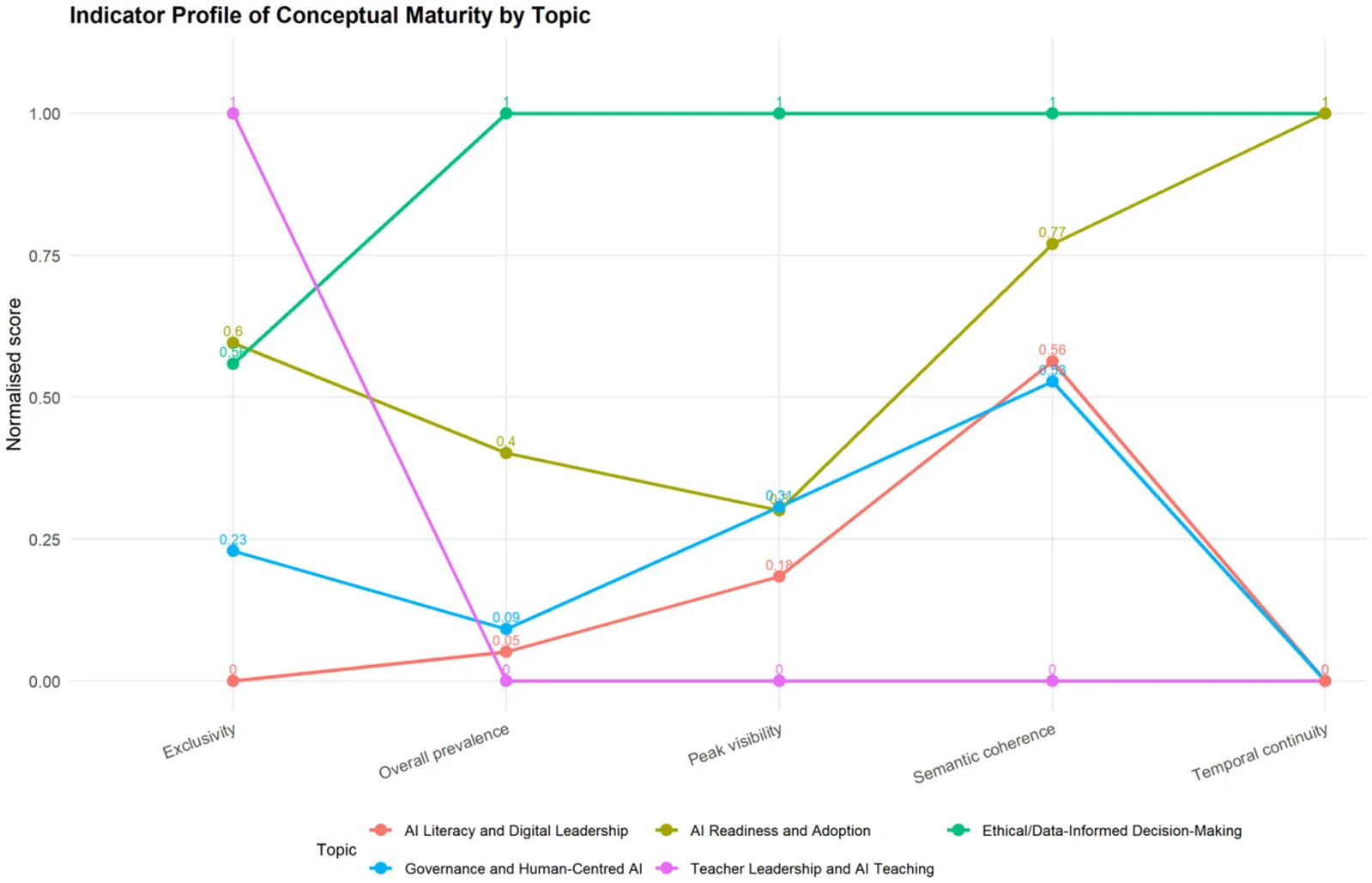
Indicator profile of conceptual maturity by topic.

Figure 4 shows the distribution of the five sub-indicators that constitute the CMI scores across the topics. This figure provides a more detailed account of which sub-indicators contribute to the overall CMI scores. In particular, the high CMI score of Topic 2 appears to stem not from a single indicator but from its strong performance across overall visibility, peak visibility, temporal continuity, and semantic coherence. By contrast, although Topic 4 reaches 1.00 in exclusivity, its remaining at 0.00 on the other indicators shows that conceptual differentiation alone is not sufficient for conceptual maturity.

Among the three themes classified at a low maturity level, Topic 5, namely “Governance Frameworks for Ethical and Human-Centered AI Leadership,” has the relatively highest value, with a CMI score of 23.1. As shown in Figure 4, this theme stands at 0.23 in terms of exclusivity, 0.09 in terms of prevalence, and 0.31 in terms of peak visibility. Its semantic coherence value of 0.53 indicates that the theme possesses a certain degree of internal coherence. However, its temporal continuity value of 0.00 indicates that this theme had the lowest relative temporal continuity among the five topics, rather than indicating the complete absence of temporal visibility. For this reason, although discussions of human-centered and ethical governance have begun to gain prominence in the literature, they have not yet developed into a sustained and strongly consolidated line of research.

Topic 4, “Teacher Leadership, Distributed Agency, and Classroom Practices in AI-Integrated Teaching,” is at a low level of conceptual maturity, with a CMI score of 20.0. The most notable characteristic of this theme is that it reaches a value of 1.00 on the exclusivity indicator. This finding shows that the axis of teacher leadership, distributed agency, and AI-supported teaching has a highly distinctive conceptual profile compared with the other themes. Yet, the same theme remains at 0.00 for prevalence, peak visibility, semantic coherence, and temporal continuity within the five-topic solution. This indicates that although the theme has distinctive, differentiated content, it is less visible, less temporally sustained, and less semantically consolidated than the other topics in this corpus. Put differently, Topic 4 is conceptually differentiated, but it has not yet matured.

Topic 3, “AI Literacy, Digital Leadership, and Educational Management Capacity,” has the lowest level of conceptual maturity, with a CMI score of 16.0. The semantic coherence value for this theme, at 0.56, indicates a degree of internal consistency. Yet, its exclusivity value of 0.00, temporal continuity value of 0.00, should be interpreted as the lowest relative positions among the five topics on these indicators, not as the absence of exclusivity or continuity. Together with its relatively low prevalence and peak visibility values, these results suggest that this theme is less clearly differentiated and less temporally sustained than the other topics in this corpus.

Viewed through the conceptual maturity analysis, the artificial intelligence literature in the field of school leadership and management exhibits an uneven and asymmetrical pattern of development. The theme of ethical and data-informed decision-making constitutes the most mature and central conceptual axis of the field. The theme of AI readiness and adoption, while strong in terms of temporal continuity and semantic coherence, exhibits a moderate maturity profile, as its visibility remains more limited. Conversely, themes such as AI literacy, teacher leadership, and human-centered governance show lower visibility, weaker continuity, and a more limited degree of integration. These findings reveal that the field does not yet possess a fully mature and evenly developed knowledge structure; rather, it is characterized by a literature that is concentrated around a particular conceptual core, while still developing in an early, fragmented, and uneven manner across certain subfields.

**RQ3**. *Do concepts in the literature on artificial intelligence in school leadership and management exhibit an integrated or fragmented semantic network structure?*

In this analysis, semantic network fragmentation analysis was conducted to examine whether the AI literature in the field of school leadership and management has developed in a conceptually integrated or fragmented manner. The analysis was conducted on the 112 articles included in the dataset and retained for the study. For concept extraction, the combined text field created from the title, abstract, keywords, and conclusion/discussion sections was used.

The dictionary-based search identified at least one retained concept in all 112 studies (Table 7). The controlled dictionary included 24 concepts. However, because “artificial intelligence” was the search-anchor concept used to construct the corpus and appeared across the included records, it was excluded from the subsequent network metrics to avoid mechanically inflated connectivity. After this exclusion, 23 analytically informative concepts were retained in the semantic network analysis. In total, 658 article–concept matches were obtained, with an average of 5.88 retained concepts per article. The full dictionary used for concept identification is reported in Appendix A.

**Table 7:** Descriptive summary of concept identification for the semantic network analysis.

Indicator	Value
Number of studies included in the analysis	112
Number of studies in which at least one retained concept was identified	112
Number of concepts in the controlled dictionary	24
Search-anchor concept excluded from network metrics	Artificial intelligence
Number of concepts retained in the semantic network	23
Total article–concept matches after excluding the search-anchor concept	658
Average number of retained concepts per article	5.88
Number of studies with no retained concept	0

Within the constructed network, the most frequently occurring concepts were school leadership, ethics, principal leadership, governance, AI readiness, and decision-making. This conceptual distribution indicates that the field is structured not only around technological innovation or tool use, but also along the axes of school leadership, ethical responsibility, governance, decision-making, leadership readiness, and professional capacity. In particular, the high frequencies of the concepts ethics, governance, and decision-making indicate that, within the literature, artificial intelligence is addressed in the context of school leadership alongside managerial and ethical responsibilities (Table 8).

**Table 8:** Frequency of retained concepts after excluding the search-anchor concept.

Concept	Concept group	Frequency
School leadership	Leadership	77
Ethics	Ethics and equity	66
Principal leadership	Leadership	57
Governance	Decision and governance	52
AI readiness	Capacity and readiness	49
Decision-making	Decision and governance	47
Professional development	Capacity and readiness	37
Equity	Ethics and equity	34
Digital leadership	Capacity and readiness	29
Organizational change	Organizational change	29
Privacy	Ethics and equity	25
Trust	Ethics and equity	25
School management	Leadership	24
Teaching and learning	Pedagogy	23
Generative AI	AI core	17
Personalized learning	Pedagogy	17
AI literacy	Capacity and readiness	14
Bias	Ethics and equity	13
Distributed leadership	Leadership	6
Instructional leadership	Leadership	6
Human-centered AI	Human-centered AI	5
Big data	AI core	4
Machine learning	AI core	2

The semantic network fragmentation analysis showed that the network maintained a single-component structure across all threshold values. At the threshold ≥ 2 level, the network consisted of 23 concepts and 184 links, with the number of components calculated as 1 and the fragmentation index as 0. When the threshold value is raised to 5, the network still maintains its single-component structure, with 19 concepts and 117 links. This indicates that the field does not become conceptually fragmented even when weaker links are gradually excluded. Put differently, the concepts are not organized as disconnected islands, but within a shared semantic structure (Table 9).

**Table 9:** Raw co-occurrence semantic network fragmentation metrics after excluding the search-anchor concept.

Threshold	Number of concepts	Number of links	Density	Number of components	Largest component	Fragmentation index	Transitivity	Modularity	Average degree
2	23	184	0.727	1	23	0	0.852	0.0278	16.0
3	21	153	0.729	1	21	0	0.841	0.0326	14.6
4	21	133	0.633	1	21	0	0.860	0.0324	12.7
5	19	117	0.684	1	19	0	0.808	0.0169	12.3

Network density and transitivity values further support the presence of a connected co-word structure among the retained concepts, although these values should be interpreted cautiously because the primary network is based on article-level co-occurrence. Density values in the network range from 0.633 to 0.729. These values indicate the presence of a highly dense co-occurrence structure among the concepts. Transitivity values are also high across all thresholds, ranging from 0.808 to 0.860. This indicates that the concepts are structured not only through dyadic relationships but also within closed triadic clusters. Thus, the concepts in the literature do not come together in a dispersed or incidental manner; rather, they form dense patterns of co-word-based semantic association.

Because article-level co-occurrence is a broad unit of analysis, an additional normalized co-occurrence check was conducted to examine whether the observed connectivity was inflated by frequently occurring concepts. For this purpose, pairwise Jaccard similarity was calculated after excluding the search-anchor concept “artificial intelligence.” This check weights each concept pair according to the proportion of shared documents relative to the total number of documents in which either concept appeared.

As shown in Table 10, the Jaccard-normalized results support a cautious interpretation of the main network. Across thresholds from 0.05 to 0.30, the retained concepts continued to form a single-component structure, although the number of concepts and links decreased as the threshold became more restrictive. This indicates that the raw article-level co-occurrence network should not be interpreted as evidence of full theoretical integration. Rather, the selected core concepts show co-word-based semantic connectivity and convergence around school leadership, ethics, governance, decision-making, AI readiness, and principal leadership.

**Table 10:** Jaccard-normalized co-occurrence sensitivity check.

Jaccard threshold	Number of concepts	Number of links	Number of components	Largest component	Density
≥0.05	22	183	1	22	0.792
≥0.10	20	130	1	20	0.684
≥0.15	19	98	1	19	0.573
≥0.20	19	72	1	19	0.421
≥0.25	16	51	1	16	0.425
≥0.30	13	31	1	13	0.397

By contrast, the very low modularity values indicate that there are no sharply differentiated subfields within the network. Modularity values in the network range from 0.0169 to 0.0326. This result indicates that although certain thematic concentrations are evident within the network, they do not constitute strongly differentiated sub-literatures. For this reason, it is more appropriate to describe the retained concept network as a connected co-word structure containing weak thematic clusters, rather than as evidence of complete theoretical integration.

The centrality analysis conducted at the ≥ 5 threshold reveals the field's conceptual core more clearly. School leadership is the most central concept in the network. This concept has a degree value of 17, a strength value of 418, and an eigenvector centrality value of 1.000. Ethics emerges as the second-strongest center, following school leadership, with a strength value of 370. Governance, decision-making, AI readiness, and principal leadership are also among the other central concepts in the network. This structure indicates that the literature has a strong conceptual core organized around school leadership, ethics, governance, decision-making, and leadership capacity (Table 11).

**Table 11:** Centrality values of the concepts.

Concept	Degree	Strength	Betweenness	Eigenvector
School leadership	17	418	79.5	1.000
Ethics	17	370	18.0	0.915
Governance	16	294	0.0	0.772
Decision-making	18	276	17.0	0.697
AI readiness	15	258	0.0	0.687
Principal leadership	15	257	0.0	0.682
Equity	16	214	0.0	0.570
Professional development	15	202	0.0	0.537
Privacy	13	154	0.0	0.422
Organizational change	12	143	0.0	0.397
Digital leadership	11	131	0.0	0.374
Trust	12	118	0.0	0.339
School management	12	114	0.0	0.330
Personalized learning	12	105	0.0	0.293
Teaching and learning	11	101	0.0	0.278
Bias	8	67	0.0	0.192
AI literacy	7	54	0.0	0.175
Generative AI	6	51	0.0	0.169
Distributed leadership	1	5	0.0	0.0147

The community detection results indicate two weak conceptual concentrations in the reduced network. The first community comprises the concepts of school leadership, ethics, governance, decision-making, equity, privacy, trust, school management, personalized learning, bias, AI literacy, and distributed leadership. This cluster represents a normative governance and ethical leadership orientation. In this context, the literature focuses on issues such as ethical decision-making, accountability, privacy, equity, trust, and institutional governance responsibility within school leadership.

The second community comprises the concepts of AI readiness, principal leadership, professional development, organizational change, digital leadership, teaching and learning, and generative AI. This cluster represents an orientation toward readiness, capacity building, and implementation. Here, the literature focuses on issues such as school leaders' preparedness, professional development, organizational change, digital leadership, and integration into teaching and learning processes. Nevertheless, given the low modularity value, it would not be appropriate to interpret these two communities as disconnected subfields. A more accurate interpretation is that these two structures represent weak thematic concentrations emerging within a connected co-word structure among the retained dictionary-defined concepts (Table 12).

**Table 12:** Community detection results.

Community	Conceptual orientation	Concepts	Average degree	Average strength
Community 1	Normative-governance and ethical leadership	School leadership; ethics; governance; decision-making; equity; privacy; trust; school management; personalized learning; bias; AI literacy; distributed leadership	12.4	182
Community 2	Readiness, capacity building, and implementation	AI readiness; principal leadership; professional development; organizational change; digital leadership; teaching and learning; generative AI	12.1	163

**Figure 5:**
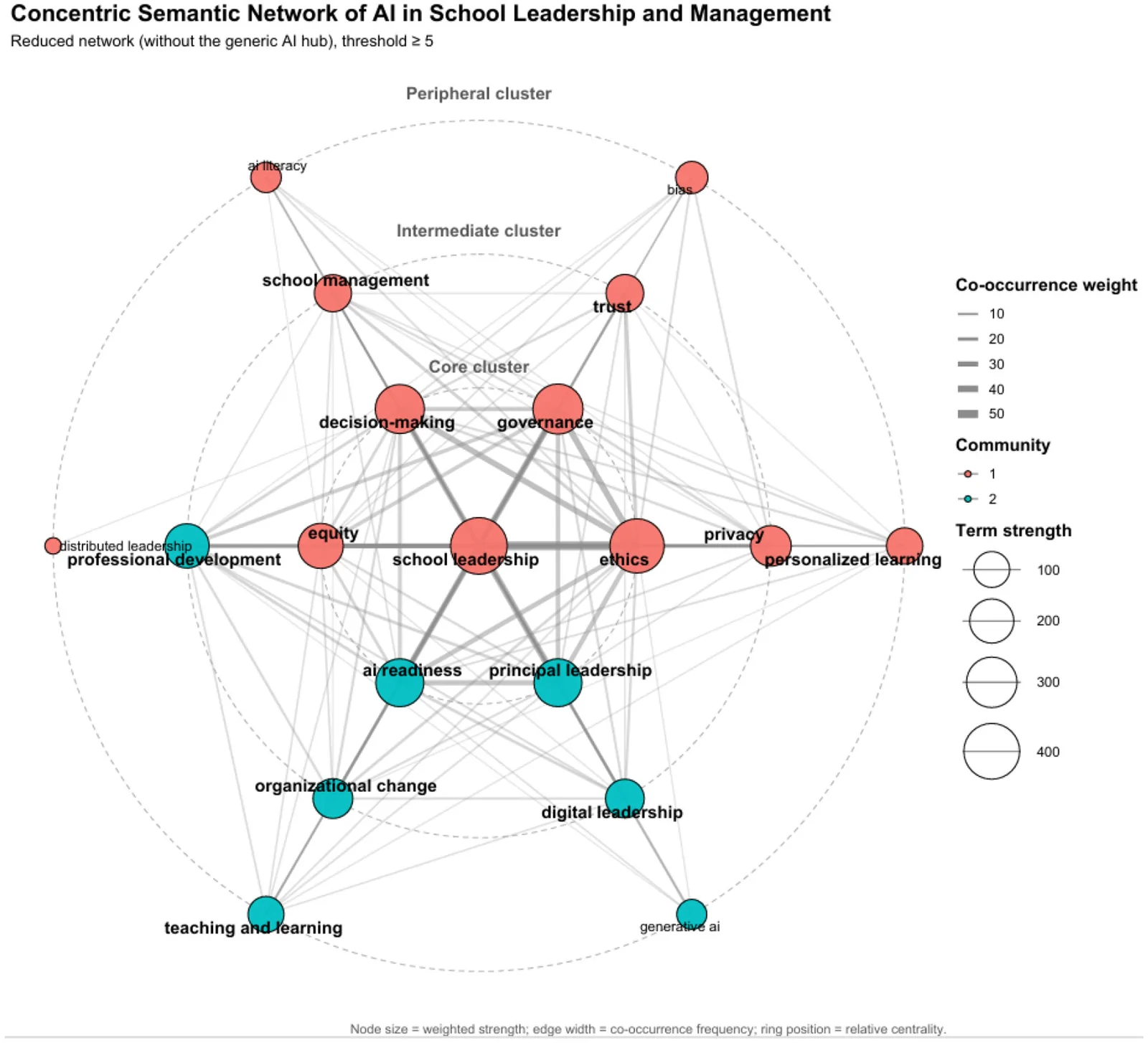
Concentric semantic network of AI in school leadership and management.

As shown in Figure 5, decision-making, governance, ethics, principal leadership, AI readiness, and equity are central concepts positioned around school leadership.

Among these concepts, ethics is the concept located closest to school leadership. In the second ring around the center, the concepts of school management, trust, privacy, digital leadership, organizational change, and professional development are positioned. The concepts of AI literacy, bias, personalized learning, generative AI, teaching and learning, and distributed leadership are located at the periphery. The colors in Figure 5 indicate the semantic communities to which the concepts belong.

The semantic network results indicate that, within the boundaries of the controlled concept dictionary used in this study, the literature on artificial intelligence in school leadership and management does not exhibit a strongly fragmented co-word structure. The network remained single-component across all threshold levels; the fragmentation index was 0 in every case, and the modularity values were very low. These findings show that although thematic diversification exists in the literature, the selected core concepts are not organized as disconnected conceptual islands. Rather, they tend to co-occur within a shared semantic space centered on school leadership, ethics, governance, decision-making, AI readiness, and principal leadership. It follows that the thematic differentiation identified in the previous STM analysis does not necessarily amount to co-word fragmentation among the core concepts captured by the dictionary. However, this finding should not be interpreted as proof that the entire field is theoretically integrated. Instead, it suggests that, despite its early-stage diversification, this literature shows signs of semantic convergence around a shared conceptual center.

## 4 Discussion

The purpose of this study is to examine, through a multilayered analytical framework, not only the themes around which the AI literature in the field of school leadership and management has developed, but also how these themes are structured, the extent to which they are differentiated, the level of conceptual maturity they exhibit, and whether they display a semantically integrated or fragmented structure. The findings show that the literature on AI and school leadership and management is expanding rapidly; however, this expansion is not unfolding as an even, linear, or fully integrated process of knowledge production, but is instead shaped around particular thematic foci, conceptual concentrations, and temporal shifts. In particular, the STM results reveal that knowledge production in the field has crystallized around five main topics: AI readiness, adoption, and self-efficacy; ethical and data-informed decision-making; AI literacy and digital leadership; teacher leadership and distributed agency; and human-centered governance. At the same time, the findings converge with some results of previous studies in the literature, extend the discussion in relation to others, and diverge from earlier findings at certain points.

First, while Tyson and Sauers (2021) examined the adoption of AI primarily in terms of leadership behaviors, interpersonal skills, and the establishment of organizational structures necessary for success, Kilinç et al. (2025) associated school principals' level of AI readiness with characteristics such as self-efficacy, a growth mindset, and openness to change. The topic “AI Readiness, Adoption, and Self-Efficacy in School Leadership” identified in this study similarly positions school leaders' readiness for AI and their capacity for adoption as central issues in the field. The semantic coherence and exclusivity findings, however, show that this theme is not merely a recurring topic but has developed into a coherent, internally distinct line of research. The study conducted by Anysiadou and Gkliati (2025) on Greek school administrators also lends support to this finding, insofar as it shows that school administrators view AI more cautiously in areas where the human element is particularly salient, such as teaching and collaboration, while regarding it as more acceptable in more technical domains, such as financial management and collaboration with service units. This indicates that AI readiness is not merely a matter of developing a positive attitude toward technology; rather, it is closely tied to how leaders distinguish between tasks that can be delegated to AI and those they regard as domains of leadership that require human judgment. In this respect, the study shows that discussions of AI readiness and adoption are no longer confined to individual leaders' characteristics or levels of openness to technology; rather, they yield a more holistic administrative profile that integrates self-efficacy, openness to change, task perception, organizational support, and leadership capacity. Still, the conceptual maturity findings, which place this theme at a moderate level, suggest that this line of inquiry has not yet reached full maturity, even though it has become clearly discernible. This interpretation is consistent with Arar et al. (2026), who regarded the adoption theme as a still-developing area.

A similar pattern is even more pronounced in the theme of ethical and data-informed decision-making. While researchers such as Wang (2021b) and Karakose and Tülübaş (2024) position the relationship between data-driven decision-making and value-based moral judgment as one of the central issues of educational leadership, Arar et al. (2026) identified decision-making, trust, big data, leadership performance, and innovation among the central clusters of the field. Dai et al. (2025) likewise conceptualized AI-supported administrative decision-making as a symbiotic relationship in which AI assumes informational roles such as data collection and analysis, while educational leaders continue to perform human and political roles such as vision building, conflict management, delegation of authority, and negotiation among stakeholders. In this study, the fact that “Ethical and Data-Informed AI Decision-Making Processes in Educational Leadership” has both the highest expected topic proportion and emerges as the strongest theme in terms of semantic coherence, exclusivity, and conceptual maturity shows a strong parallel with this body of literature. Unlike previous studies, however, this study presents ethics and data-driven decision-making not as two separate domains or as two counterbalancing poles, but as integrated components of the same leadership practice. While Polat et al. (2025) treated ethics, data privacy, security, and bias mainly within the challenge cluster, Igbokwe (2024) discussed risks such as bias, privacy, transparency, accountability, explainability, and dehumanization as areas of responsibility for educational leaders. The findings of this study, by contrast, show that ethics is not merely a domain of risk, but has become the most coherent, differentiated, and mature axis of knowledge production in the AI literature on school leadership and management. Put differently, although ethical issues and decision-making have previously been treated in the literature as two distinct lines of inquiry, knowledge production in the field now indicates that decision-making has evolved into a single integrated theme that must be understood within the framework of ethical principles.

The other three themes appear to have a more complex relationship with the literature. Ghamrawi et al. (2024) showed that AI is discussed as a force that both expands and constrains teacher leadership and teacher agency, while Berkovich (2025) demonstrated that AI-supported instructional leadership has become more visible than other areas of leadership. In this study, the fact that the theme “Teacher Leadership, Distributed Agency, and Classroom Practices in AI-Integrated Teaching” is differentiated from the other themes yet lacks internal coherence can be explained by this dual, at times tension-laden, positioning in the literature. AI is discussed, on the one hand, as a tool that strengthens teachers' professional agency and supports personalization, curriculum development, and professional learning, and, on the other hand, as a mechanism of control that may narrow teacher autonomy and turn teachers into implementers of algorithmic decisions. Bixler and Ceballos's (2025) principal–AI use model supports this theme by showing that school principals can use AI in areas such as data analysis, self-directed learning, evaluating possible outcomes, and developing instructional leadership plans. However, while this model focuses primarily on the principal's use of AI for instructional leadership, the finding on distributed agency in the present study suggests that leadership should be reconsidered not only as a principal-centered practice, but also as a collective domain of agency distributed among teachers and other actors within the school.

Similarly, while Sposato (2025) classified areas such as administrative efficiency, organizational leadership, strategic planning, ethical leadership, and governance under separate headings, Kafa (2025a,b) showed that AI tools can support tasks in school management such as communication, administrative efficiency, decision-making, data processing, and scenario planning. Yet the relative weakness of the themes “AI Literacy, Digital Leadership, and Educational Management Capacity” and “Governance Frameworks for Ethical and Human-Centered AI Leadership” in terms of both internal coherence and conceptual differentiation indicates that concepts such as digital leadership, AI literacy, governance, and human-centered AI still constitute emerging, fragmented, and conceptually unsettled areas of inquiry within the literature. The analysis by Richardson et al. (2025) of the messages conveyed about AI in practitioner-oriented publications for school leaders during the first year following the public release of ChatGPT also supports this fragmented structure, as the recommendations offered to school leaders often revolve around capacity building, curiosity, experimentation, risk awareness, and local decision-making processes, yet do not produce a conceptually established framework for AI leadership. Nevertheless, although the governance theme was classified as a low-maturity theme in the present corpus, its visibility and internal coherence suggest, in line with the emphases placed by Arar et al. (2025), Polat et al. (2025), Sposato (2025), Karakose (2024), and Karakose and Tülübaş (2025) on ethics, regulatory frameworks, human-centered AI, Leadership 4.0, and Education 4.0, that this area has the potential to develop into a more institutional and structural line of inquiry in the near future.

The semantic network fragmentation analysis, however, provides a more nuanced result that partially challenges the literature's emphasis on a fragmented knowledge structure. The apparent tension between the low conceptual maturity of some STM topics and the integrated structure observed in the semantic network should be understood as a difference in analytical level. The former refers to the internal consolidation of latent themes, whereas the latter refers to co-word-based connectivity among selected core concepts. Thus, the findings point not to a fully integrated theoretical field, but to an unevenly developed thematic structure that nevertheless shares a connected semantic core. For example, Kilinç et al. (2025) argue that the literature on principal readiness for AI use is largely descriptive and fragmented, with some studies focusing on strategic and operational benefits and others on ethical, relational, and equity-based concerns. Similarly, Sposato (2025) argued that the applications of AI in educational leadership have produced fragmented adoption practices because of the absence of a comprehensive framework, while Kafa (2025a,b) and Richardson et al. (2025) also showed that the AI literature in the context of school leadership remains limited, developing, and characterized by dispersed practitioner-level recommendations. By contrast, the semantic network fragmentation findings of this study show that the AI corpus in the field of school leadership and management has not come together in a random, disconnected, or dispersed manner; rather, dense patterns of co-word-based semantic association exist among the retained concepts. Arar et al.'s (2026) identification of multiple clusters around leadership, decision-making, trust, innovation, optimization, prediction, and future work practices in their keyword co-occurrence analysis likewise indicates that the field contains different focal points, yet that these focal points are not entirely disconnected from one another. The two main lines of inquiry identified in this study make this structure more explicit: the first is shaped around a normative-governance and ethical leadership axis, while the second focuses on readiness, capacity building, and implementation processes. This finding, therefore, does not entirely reject the characterization of the literature as “fragmented”; rather, it suggests that fragmentation should be understood not as random disconnection, but as an early-stage semantic differentiation organized around two complementary lines of inquiry. The Jaccard-normalized check further supports this cautious interpretation by showing that semantic connectivity should be understood as relative conceptual proximity among selected core concepts, not as a strong claim of theoretical integration across the entire field.

In synthesis, this study addresses an important gap in the literature by bringing together findings from multiple analyses to explain how the AI literature in the field of school leadership and management is thematically structured, the extent to which these themes have attained conceptual maturity, and whether the core concepts in the field exhibit an integrated or fragmented structure. Although previous studies have discussed the effects of AI on school leadership and management, ethical risks, readiness, digital leadership, or governance issues separately, this study evaluates this seemingly dispersed knowledge domain within a single analytical framework through three complementary analyses. In doing so, the study moves beyond merely showing which themes exist in the field and demonstrates which themes actually constitute coherent and differentiated lines of knowledge, which have not yet reached conceptual maturity, and whether the concepts in the field are fragmented. In this respect, the significance of the study lies not merely in describing the AI and school leadership and management literature as a growing field of inquiry, but in showing the axes along which this growth has become institutionalized, the areas in which conceptual ambiguity still persists, and the theoretical and methodological gaps to which future research should be directed. The study therefore does more than map the current state of the field; it also proposes a more holistic and empirically grounded research agenda for ethical and data-informed decision-making, AI readiness, human-centered governance, and context-sensitive leadership research.

## 5 Conclusions

The STM analyses conducted in this study reveal that the AI corpus in school leadership and management is clustered under five topics: “AI Readiness, Adoption, and Self-Efficacy in School Leadership”; “Ethical and Data-Informed AI Decision-Making Processes in Educational Leadership”; “AI Literacy, Digital Leadership, and Educational Management Capacity”; “Teacher Leadership, Distributed Agency, and Classroom Practices in AI-Integrated Teaching”; and “Governance Frameworks for Ethical and Human-Centered AI Leadership.” The topic with the highest expected topic proportion is “Ethical and Data-Informed AI Decision-Making Processes in Educational Leadership.” The second highest topic is “AI Readiness, Adoption, and Self-Efficacy in School Leadership.”

In the early stages of the field, knowledge production appears to have emerged around ethical and data-informed decision-making. As of 2024, the topic “AI Literacy, Digital Leadership, and Educational Management Capacity” became strongly visible. In 2025, the main focus of the literature shifted to “AI Readiness, Adoption, and Self-Efficacy in School Leadership”; however, “Ethical and Data-Informed AI Decision-Making Processes in Educational Leadership” maintained its strong position in the same year. In 2026, the topic “Governance Frameworks for Ethical and Human-Centered AI Leadership” began to come to the fore. These findings reveal that ethics in the use of artificial intelligence in school leadership has remained present from the earliest studies in the field to the most recent ones, and that many researchers have contributed to knowledge production on the ethical use of artificial intelligence.

The semantic coherence and exclusivity values identified in the STM analysis show that the themes “AI Readiness, Adoption, and Self-Efficacy in School Leadership” and “Ethical and Data-Informed AI Decision-Making Processes in Educational Leadership” have developed into internally coherent themes that are clearly differentiated from the other topics. These two themes, which have persisted since the early stages of the field, appear to have achieved a semantically coherent profile. Although the theme “Teacher Leadership, Distributed Agency, and Classroom Practices in AI-Integrated Teaching” is conceptually differentiated from the other themes, it has not yet achieved internal coherence. By contrast, the themes “AI Literacy, Digital Leadership, and Educational Management Capacity” and “Governance Frameworks for Ethical and Human-Centered AI Leadership” have not yet attained either internal coherence or conceptual differentiation. The conceptual maturity analysis also shows that the theme “Ethical and Data-Informed AI Decision-Making Processes in Educational Leadership” was classified as a high-maturity theme, while “AI Readiness, Adoption, and Self-Efficacy in School Leadership” was classified as a moderate-maturity theme. The remaining three themes, including “Governance Frameworks for Ethical and Human-Centered AI Leadership,” were classified as low-maturity themes.

The semantic network fragmentation analysis shows that the retained dictionary-defined concepts are not dispersed or random at the level of article-based co-word association; rather, they exhibit a connected pattern of semantic convergence. The analysis shows that two lines of inquiry have emerged. The first focuses on normative governance and ethical leadership, while the second centers on readiness, capacity building, and implementation. This finding overlaps with the content of the two dominant and mature themes identified in the STM analyses.

This finding should be interpreted together with the conceptual maturity results rather than as a contradiction to them. The STM and conceptual maturity analyses show that the field is unevenly developed at the thematic level, with some topics still lacking strong coherence, continuity, or differentiation. By contrast, the semantic network analysis shows that the selected core concepts are not disconnected from one another, but tend to co-occur within a shared semantic space. Therefore, the study does not suggest that the field is fully theoretically integrated. Rather, it indicates that AI research in school leadership and management is still developing unevenly across themes, while showing signs of co-word-based semantic convergence around shared concepts such as school leadership, ethics, governance, decision-making, AI readiness, and principal leadership.

In conclusion, the AI corpus in school leadership and management was found to have an intellectual foundation organized into thematic clusters across five main topic areas. Two of these five topic areas were found to possess conceptual maturity, semantic internal coherence, and distinctive differentiation, whereas the remaining three areas are still at a developmental stage, although they also display a certain degree of semantic internal coherence and exclusivity. Despite this differentiation, within the boundaries of the controlled dictionary and at the level of article-based co-word association, the retained concepts in the AI corpus on school leadership and management were found not to exhibit a strongly fragmented or dispersed structure; rather, they formed a connected co-word structure in which conceptual distinctions have begun to crystallize. More specifically, the semantic network analysis suggests that the selected core concepts are not organized as disconnected conceptual islands. After excluding the search-anchor concept and applying a Jaccard-normalized check, the retained concepts continued to show co-word-based semantic convergence around a shared conceptual center. Therefore, this conclusion should be understood not as a claim of complete theoretical integration, but as evidence that an unevenly developing thematic field is nevertheless organized around a connected semantic core.

## 6 Limitations and future directions

The three-analysis design of this study enables evaluation of the thematic, conceptual, and semantic structures of the AI literature in the field of school leadership and management in conjunction with one another; however, each analysis has certain limitations. First, although Structural Topic Modeling makes the dominant themes in the articles visible, it does not provide a fully mechanical classification, since topic labeling requires researcher interpretation. In addition, although some articles contribute to more than one theme, the model represents them through their dominant topic. Second, the conceptual maturity index comparatively indicates the level of conceptual maturity in the field; however, it risks reducing the qualitative richness of theoretical debates in the literature to numerical indicators. Finally, semantic network fragmentation analysis indicates the degree of integration or fragmentation among concepts; however, the co-occurrence of concepts does not necessarily imply a strong theoretical relationship between them. In addition, because the analysis relied on a controlled dictionary of 24 concepts, the findings should be interpreted as applying to the selected core concepts rather than to the entire conceptual universe of the field. Nevertheless, this dictionary-based strategy was appropriate for the purpose of the study, which was to examine whether theoretically central concepts in AI and school leadership research formed an integrated or fragmented co-word structure. Moreover, because the primary network was based on article-level co-occurrence, some links may reflect broad topical co-presence rather than direct conceptual relationships within the same paragraph or argument. Although a Jaccard-normalized check was used to reduce the influence of highly frequent terms, future studies may further refine the analysis by using paragraph-level co-occurrence, sentence-level co-occurrence, or embedding-based semantic similarity.

These limitations point to a concrete research agenda for future studies. First, the relatively higher conceptual maturity of the themes “Ethical and Data-Informed AI Decision-Making Processes in Educational Leadership” and “AI Readiness, Adoption, and Self-Efficacy in School Leadership” also provides a promising direction for future empirical research. However, because the present study is a text mining-based computational literature review rather than a construct validation study, these themes should not be treated as ready-made measurable variables. Future studies may build on these findings by developing clearer conceptual definitions, operational indicators, and validated measurement tools for these themes. After such conceptual and measurement work, researchers may examine how ethical and data-informed AI decision-making relates to instructional leadership, teachers' trust in school administrators, school climate, organizational wellbeing, or other established constructs in educational leadership and management. Next, researchers should extend the AI literature not only through technological adoption or school leaders' levels of AI readiness, but also by linking it to the established areas of inquiry in educational management, administration, and leadership. For example, instructional leadership research can be reconsidered through AI-supported instructional decision-making, learning analytics, and algorithmic systems that support teacher development. Studies on distributed and shared leadership may examine the effects of human–AI collaboration on teacher autonomy, professional learning communities, and the distribution of leadership. In the areas of ethical leadership, data-informed decision-making, and social justice leadership, issues such as algorithmic transparency, data privacy, the risk of discrimination, and accountability should be examined more rigorously. In addition, to mitigate the influence of researcher subjectivity, future network analyses may be conducted using methods such as exponential random graph modeling, decision trees, and neural network-based analysis. In areas where semantic internal coherence has not yet been established, such as “Teacher Leadership, Distributed Agency, and Classroom Practices in AI-Integrated Teaching,” there appears to be a need to develop greater conceptual coherence among researchers. Researchers are advised to conduct experimental studies on the transfer of AI applications into classroom practice and, in the process, examine how roles and responsibilities should be distributed among school leaders, administrators, teachers, parents, and students. Similarly, it appears that the concept of AI leadership in school leadership and management is likely to gain a theoretical foundation in the near future and establish a distinctive position within the leadership literature. In this context, we recommend conducting conceptual studies on AI leadership in educational management and beginning to delineate the theoretical framework of this emerging leadership theme.

For school leaders and administrators, the primary agenda is to view AI tools not merely as technical instruments that enhance efficiency but as administrative decision-support systems with pedagogical and ethical implications. For this reason, schools should develop clear data policies on the use of AI, provide AI literacy training for teachers, ensure human oversight in decision-making, and establish institutional protocols that protect students' rights. In addition, to promote the ethical use of artificial intelligence and make it more human-centered, it is recommended that discussions move beyond theoretical debates, that AI be incorporated into instructional practice, and that good practices be made more visible.

From the perspective of policymakers, the most concrete need is the development of national or local guidelines regulating the use of AI at the school level. These guidelines should cover principles related to data security, algorithmic bias, leadership training, teacher support, and equitable access. Future research should particularly expand longitudinal studies, comparative cross-country analyses, school-based case studies, mixed-methods research, and intervention designs. In this way, the field can move beyond early-stage conceptual dispersion and provide a more holistic account of the effects of AI on school leadership, management, ethical decision-making, instructional improvement, and educational equity.

## Data Availability

The final list of included studies, data-extraction framework and variable definitions, citation matrix or edge list, article-level analytical variables, and R scripts used in the analyses are available from the corresponding author upon reasonable request.
